# Investigation on CuO nanoparticle enhanced mahua biodiesel/diesel fuelled CI engine combustion for improved performance and emission abetted by response surface methodology

**DOI:** 10.1038/s41598-024-77271-3

**Published:** 2024-11-06

**Authors:** Sinnappadass Muniyappan, Ravi Krishnaiah

**Affiliations:** School of Mechanical Engineering, VIT University, Vellore, 632014 Tamil Nadu India

**Keywords:** Mahua biodiesel, CuO as combustion catalyst, Mahua biodiesel-diesel fuel concoction, RSM prediction and optimization of CI engine parameters, CI engine performance, combustion and emissions characteristics improvement., Environmental sciences, Energy science and technology, Engineering

## Abstract

In this study, the characteristics of diesel engines were tested with in-house produced mahua biodiesel blended with diesel and copper oxide nanoparticles (CuO NP) catalyst. The preliminary investigation used mahua biodiesel-diesel blends (M10, M20, and M30) among them M20 outperformed. Further M20 and CuO NP with concentrations of 25, 50, and 75 ppm are studied. Finally, the response surface methodology (RSM) was used to determine the appropriate NP concentration for M20. The findings showed that the blend of M20 with 60 ppm NP at 80% load had the highest desirability (0.9740), and the developed RSM model predicted engine responses with a mean absolute percentage error (MAPE) of 3.0962% to the confirmation test confirming the model’s accuracy. The optimized M20NP60 blend demonstrated superior combustion, performance and emission characteristics.

**Supplementary Information:**

The online version contains supplementary material available at 10.1038/s41598-024-77271-3.

## Introduction

The air quality has reduced significantly due to increasing vehicle emissions, including HC, CO, and NOx, mainly from the transportation sector resulting in health issues and environmental degradation worldwide^1^. Diesel engines are essential in all transportation, industry, and agriculture sectors because of their superior thermal efficiency, high output power, durability, and reliability. But diesel engines have been plagued with harmful pollutants. Due to the increasing depletion of diesel fuel, organisations, consumers, and academics researched renewable alternatives. The diesel engine’s smoke and NOx reduction is another major challenge for researchers^2^. The primary source of hazardous pollution emissions from the automobile industry is the burning of fossil fuels; these emissions lead to the formation of acid rain, greenhouse gases, smog, and undesirable alterations in climate^3^. The rapid depletion of non-renewable fuels caused the need to investigate and create alternative renewable fuels. Vegetable oils are considered a potential alternative energy source to replace fossil fuels in this situation. Due to similar qualities to diesel fuel, biodiesel has gained as an alternative fuel for diesel engines^4^. Sustainable alternative fuels are gaining attention due to fossil fuel depletion, biodiesel is a superior alternative fuel for diesel engines. Plants, algae, animal fat, vegetable oils, waste oil, etc., may all be used to make biodiesels. Biodiesels derived from vegetable and animal fats are safe and biodegradable^5^. Biodiesel is very close to petroleum fuels in terms of its characteristics and chemical structure. Conversely, biodiesels lack sulphur, polycyclic aromatics, or other dangerous chemicals^6^. The main advantage of using biofuels is that they are easily available, renewable, and beneficial to the environment. Biofuels exhibit greater combustion quality compared to regular diesel fuel as a result of their increased oxygen (O_2_) content^7^.

The feedstocks for the first generation of biodiesel were lipids derived from animals and plants, whereas the second generation used byproducts from forests and farms. High demand for edible oils as food sources may cause scarcity and economic inequality. Second-generation feedstock nonedible sources has environmental and economic advantages that encourage researchers to overcome obstacles in producing biodiesel from first generation feedstock^8^. This study examines Mahua seed biodiesel. The oil is native to India and grown in drought-prone and warm environments. One kilogram of seed generates 250 cc of oil. The kernel is extracted by drying the seeds, crushing them in village ghanis, expeller pressing, and solvent extraction yielding 40–43% of oil. Methyl esters from free fatty acids (FA) are used as biodiesel^9^. Mahua oil (MO) can be utilized as a substitute fuel for diesel engines because of its superior properties, such as cetane number (CN) and calorific value (CV), which are comparable to diesel fuel^10^. MO is a great feedstock, since it is readily accessible, inexpensive, and has a high oil extraction quality compared to other feedstocks in the market. Furthermore, the biodiesel is produced from crude MO through an alkaline-based transesterification process employing potassium hydroxide as a catalyst. In contrast, biodiesel produces lower levels of HC, CO, and NOx. Results in reduced BTE compared to diesel^11^.

Adding NP to biodiesel blend resulted in higher BTE and greatly dropped in CO, HC, and smoke^12^. When NP are present, their catalytic activity increases, leading to better combustion and a shorter ignition time (ID). Additionally, NP reduce the metal temperature and thermal loading of the combustion chamber, as well as the heat flow^13^. During combustion, the presence of NP can enhance the surface-area-to-volume ratio (SA/V) and boost the BTE, resulting in enhanced air-fuel mixing and more thorough combustion^14^. NP have more surface area, which increases fuel oxidation and combustion. Using the NP additions for the biodiesel blend can improve outcomes. In one of the study, NP were added to diesel to improve ignition. Diesel NP concoctions always ignited better than diesel^15^. Biodiesel-diesel blends with varying amounts of NP reduced undesired emissions such as HC, CO, and smoke while increasing NOx and CO_2_. Biodiesel with NP consistently improves performance by enhancing BTE and reducing BSFC^16^. Several researchers have recently published studies on the influence of various nano additions on diesel engines to assess their performance, emissions, and combustion characteristics while operating on different biodiesels. According to a previous study, adding NP (alumina, titanium di-oxide, and Cerium di-oxide) to diesel and biodiesel fuels can greatly increase the BTE while reducing CO, smoke, BSFC, and HC compared to using biodiesel alone^17^. The performance and emissions of the CI engine were examined in an experiment using 20% Mahua biodiesel combined with titanium di-oxide NP. Compared to diesel, the outcome indicated lower emissions and improvements in BTE^18^. Sathish et al., observed that the metallic NP were utilised as effective catalysts for combustion in CI engines, resulting in a substantial reduction in emissions and an improvement in engine performance^19^. To reduce the ID and ensure efficient engine performance of biodiesel and diesel fuel blends, several studies have favored the use of metal-based NP^20^. Zinc oxide NP added to algal oil biodiesel enhanced BTE while lowering EGT, NOx, HC, CO, and smoke. Using zinc oxide NP, biodiesel-diesel blends resulted in more BTE and lesser CO, HC, and smoke. The researchers discovered that adding Zinc oxide NP to diesel-biodiesel blends increased net heat release (HRR) and peak cylinder pressure (CP)^21^. Addition of aluminium oxide NP with 20% Mahua biodiesel blend and evaluated experimentally engine performance and emissions to achieve favourable outcomes^22^. Adding NP like titanium di-oxide and Zirconium di-oxide will improve oxidation, lower the exhaust gas temperature, and increase the base fuel’s surface area. This will improve burning and lower emissions^23^. Ooi et al., examine the experimental investigation of multi walled carbon nanotube blended with palm oil biodiesel resulted in higher BTE and lower CO, HC and more NOx^24^. The authors investigate the effects of graphite oxide and single walled carbon nanotube with cerium oxide to enhancing the combustion characteristics and greatly reduce the CO and HC^25^. Simhadri et al., carried out research using cerium di-oxide NP at different concentrations of 25, 50, 75, and 100 ppm blended with Mahua biodiesel B20. B20 was found to have greater BSFC and less BTE when compared to diesel in terms of performance and combustion behaviour. Nevertheless, compared to B20, there was a 1.9% increase in BTE and a 3.8% decrease in BSFC when B20 with 25 ppm cerium di-oxide was injected at 240 bar timing. A 200 bar injection time produced greater CP and lower HRR than diesel^26^. The authors investigated the effects of using CuO as a NP in mahua oil bio-diesel (BD-100). They verified that while BTE has been shown to be less than that of diesel, BSFC of BD-100 was shown to be greater than that of diesel^27^. Murugesan et al., examine the effect of graphite oxide along with algae biodiesel to enhance the performance characteristics and reduce the NOx^28^. Jatropha biodiesel (JB20) was blended with 75 ppm CuO NP, resulting in increased BTE, decreased BSFC, and a significant reduction in CO, HC, and smoke^29^. Pumpkin seed oil methyl ester diesel (B20) is blended with varying concentrations of CuO NP (50 and 100 ppm). The B20CuO100 blend resulted in 2.3% more BTE than the B20 blend, but reduced BSFC by 6.4%. Engine HC, CO, and smoke emissions were considerably reduced when CuO NP blended with B20. The addition of CuO NP to B20 improved combustion properties including HRR and cylinder pressure^30^. Palm oil biodiesel blended with CuO NP resulted in 1.6% higher BTE, 6.7% lower BSEC, and greatly dropped in CO, HC, smoke except NOx^31^.

Previous studies found that the use of nano-additives significantly enhanced stability and other physicochemical parameters of fuels. The addition of certain amount of CuO NP to a diesel-biodiesel blend reduced the blend’s density and viscosity. Incorporating CuO NP into fuel samples reduced their flash and fire points while increasing their CV. Nevertheless, a drawback of employing biodiesel is its lower heating value and poor cold flow characteristics, which might prevent the improvement of efficiency. To address this problem, nanoparticle additions provide a better remedy for enhancing these properties of biodiesel. The presence of CuO NP in the biodiesel blend promotes the formation of micro-explosions during combustion, resulting in enhanced air and fuel mixing, complete combustion, and a higher BTE. The CuO NP thermal transmissibility have shown to accelerate the fast chain reactions during combustion. As a consequence of the shorter ignition delay (ID) and total fuel evaporation time, the rate of combustion has increased. Because of the available O_2_ storage capacity, improved catalytic activity of CuO NP encouraged more efficient and complete combustion, resulting in an improvement in BTE^32^. Higher NP concentrations increased the availability of active contact surfaces and boosted the catalytic impact of NP^33^. It has been observed that NP improved atomisation and exceptional blending capacity leading to enhanced fuel combustion.

There is a significant time and money loss associated with conducting complex experiments to determine engine performance and emission characteristics. Numerical and statistical approaches by modelling and predicting engine parameters had helped the researchers to overcome this issue. A variety of optimisation methods such as RSM, ANN, Taguchi, GA, and others were applied sequentially to get engine operating parameters in order to reduce the number of engine experiments^34^. In creating a prediction model, engine inputs such as injection parameters, engine speed, load, fuel mix, etc., and outputs such as BTE, BSEC, BSFC, peak CP, and emission indices were used. To optimise the parameters based on desirability, the specific models are employed. Optimising output factors and engineering-based models using test variables is another common use case of RSM. By designing a suitable test matrix and reducing the total number of tests, RSM optimises the process in the least amount of time compared to other methods. In one research, the optimal diesel-biodiesel blend is obtained utilising multi-response optimization using RSM model linked with CCD. The BTE, BSFC, CO, HC, and NOx are predicted for the engine reactions with less than 5% error^35^. In a study for enhancing the performance in a tobacco biodiesel fuelled engine, the input parameters used included biodiesel blends, engine load, injection pressure, injection time. With in the ideal combination of the parameters falling into predicted ranges, the engine’s performance was improved^36^. The optimisation of process parameters in a diesel engine was examined using various alcohols (DEE, Ethanol) and biodiesel (palm oil) blends were assessed for efficiency, emission, and combustion properties using RSM approach. Engine parameters for optimisation included the blend ratio and engine load to reduce BSFC, HC, CO, and NOx and increase BTE and peak pressure^37^. In a study, to create connection functions between the input factors (loads and blend ratio) and the engine output responses (combustion, performance, and emissions), the variance analysis approach was used. Using RSM optimisation, the researchers expect to find the ideal response values and related parameters for optimising the experimental process. Empirical data supporting the analysis of diesel engine combustion characteristics under Central Composite Design (CCD) modes consists of metrics relating to combustion (peak CP and HRR), performance (BTE and BSFC), and emission (CO, HC, NOx, and Smoke). The most important output parameters are found in the research by use of ANOVA analysis and RSM model. RSM technique are known to be used in the validate the engine efficiency and emission parameters.

Based on the literature review it is concluded that no research exist on the experimental investigation and novel RSM approach for optimizing the diesel engines fueled by blends of mahua biodiesel, CuO NP with less number of trails. This study considered D100 (diesel), M20 (20% mahua oil biodiesel + 80% diesel), M20NP25 (20% mahua oil biodiesel + 80% diesel + 25 ppm of CuO NP), M20NP50 (20% mahua oil biodiesel + 80% diesel + 50 ppm of CuO NP), and M20NP75 (20% mahua oil biodiesel + 80% diesel + 75 ppm of CuO NP) for investigation.

The novelty of this study is use of RSM and CCD as a tool for predicting intermediate experimental results and optimising performance and emission by mathematically modelling the tool by providing different weightages on the independent variables and interaction between them. This resulted in less number of trials, thereby saving time and money.

## Materials and methods

**Figure 1:**
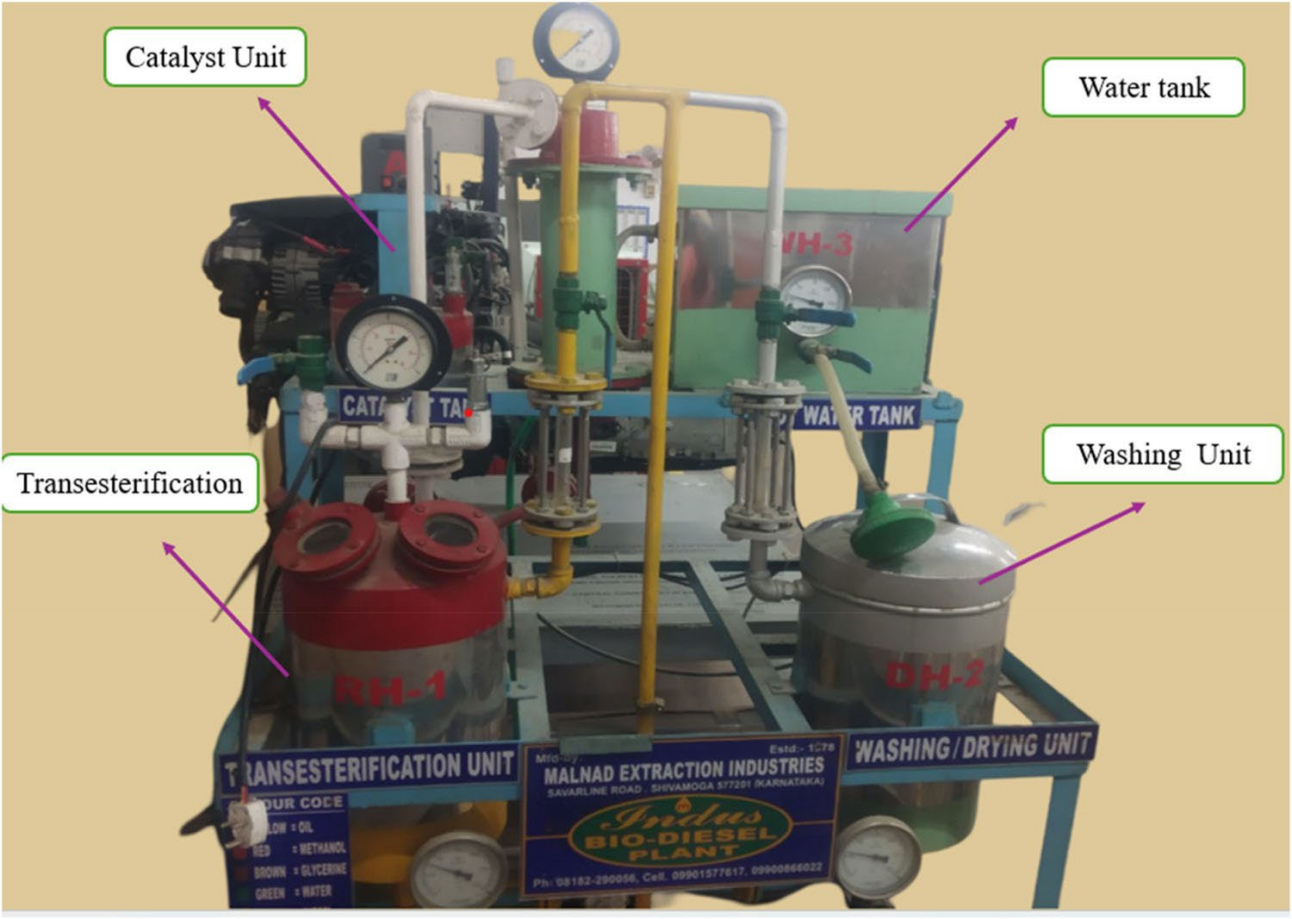
Production of mahua oil biodiesel.

The preparation of M100 from raw oil (Agro Biotech, India) involves a base-catalyzed transesterification process as show in Fig. 1. The purity of Sulphuric acid – 98% (H_2_SO_4_) and sodium hydroxide – 80% (NaOH) used for this process. The crude oil was heated to a temperature of 70 °C and then mixed with a solution comprising catalyst and alcohol. The mixture’s temperature is increased by 5 °C until all the methanol evaporates, separating glycerol and ester. Table 1 shows the FA composition of mahua oil.

**Table 1:** FA content of mahua oil.

S.no	Fatty content	Component name	Formula	Structure numbers	Wt.(%)
1	Palmitic acid	Hexadecanoic	C_16_H_32_0_2_	16:0	16.0–28.2
2	Stearic acid	Octadecanoic	C_18_H_36_0_2_	18:0	20.0–25.1
3	Arachidic acid	Eicosanoic	C_20_H_40_0_2_	20:0	0.0–3.3
4	Oleic acid	cis-9-Octadecenoic	C_18_H_34_0_2_	18:1	41.0–51.0
5	Linoleic acid	cis-9,cis-12-Octadecadienoic	C_18_H_32_0_2_	18:2	8.9–13.7

**Figure 2:**
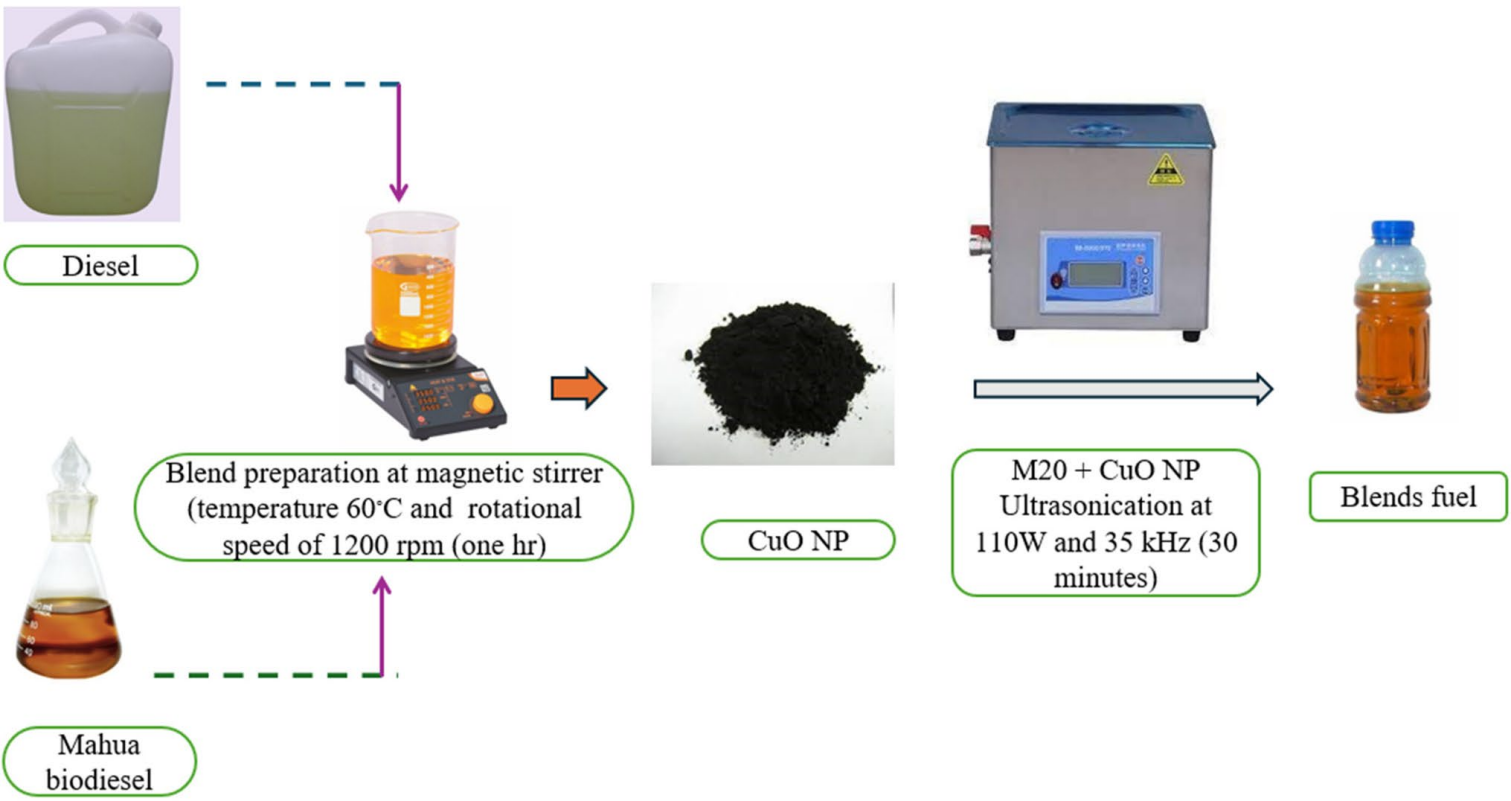
NP blending in M20 fuel blend through ultrasonicator.

Sigma-Aldrich in India delivers 99.9% pure CuO NP. The nanoparticles ranged in size from 30 nm to 40 nm. Table 2 illustrate the properties of CuO NP were selected for this study because of their increased heat capacity, melting temperature, thermal expansion, and thermal heat conductivity compared to other NP. CuO is superior to other NP due to its non-toxic synthesis, sustainability, and rapid production. To make Mahua biodiesel from crude oil, the transesterification process was used. They purchased diesel and CuO NP already on the market to make nano fuel blends. This investigation used a 20% biodiesel blending with 80% diesel, denoted by M20 blend, as fuel. The concentrations of NP used to make the nano fuel blends varied from 25 to 75 ppm. After 4 weeks, researchers observed that nano fuel blended with 25, 50, and 75 ppm were stable^38^. Figure 2 shows the CuO NP were added at 25, 50, and 75 ppm dosages to each blend M20 using an ultrasonicator. The ultrasonication method is the best way to disperse the NP in a base fluid because it allows the possible agglomeration of NP to return to the nanoscale range. The NP were initially weighed to a quantity of 25 ppm and then dispersed throughout all of the biodiesel blends with the aid of an ultrasonicator set to a power and frequency of 110 W and 35 kHz, respectively, for 30 min. This process produced NP mixed with biodiesel fuel (M20 + 25 ppm). To determine the stability qualities, a technique similar to the one described above investigates the 50 and 75 ppm CuO NP introduced to biodiesel fuels and put in test tubes under stable settings^39^. Tables 3 and 4 shows the properties of test fuel characteristics.

**Table 2:** Properties of CuO Nanoparticle.

S.no	Property	CuO
1	Chemical name	Copper oxide
2	Chemical composition	Copper − 79.9%, oxygen − 20.1%
3	Molecular weight	32.85 g/mol
4	Particle size	20–30 nm
5	Colour	Pure black
6	Thermal conductivity	0.732 W/m-K
7	Density	6.31 g/cm^3^
8	Melting point	1201 °C
9	Molar mass	79.5 g/mol
10	Boiling point	2000 °C

**Table 3:** Properties of diesel and mahua oil biodiesel.

Properties	Diesel D100	M100
Viscosity (mm^2^/s)	2.54	3.98
Density (kg/m^3^)	837	880
Cetane Index	50	54
Flashpoint (˚C)	42	157
Fire point (˚C)	68	185
Calorific value (kJ/kg)	43,500	41,200
Auto ignition temperature (˚C)	240	420
Latent heat of vapourization (kJ/kg)	282	361
Stoichiometric ratio A/F	0	14.5

**Table 4:** Test fuel characteristics.

Properties	D100	D80M20	D80M20NP25	D80M20NP50	D80M20NP75
Viscosity (mm^2^/s)	2.54	2.68	2.71	2.73	2.77
Density (kg/m^3^)	837	850	853	855	858
Cetane Index	51	48.2	50.4	52	51.3
Calorific value (kJ/kg)	43,200	42,430	43,460	44,534	43,831

## Experimental setup

**Figure 3:**
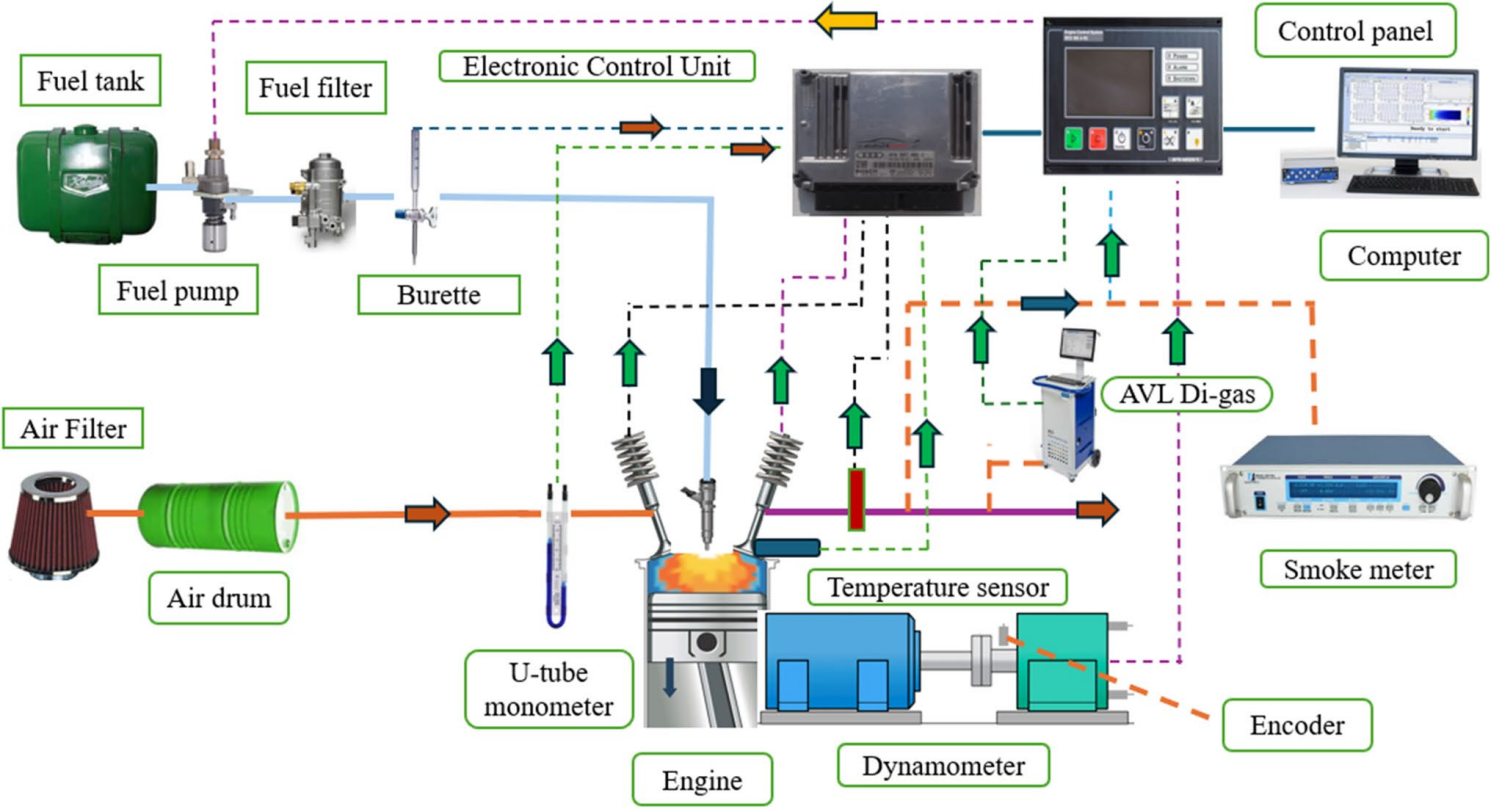
Single-cylinder 4-stroke VCR diesel engine test setup.

Engine characteristics were tested on a VCR engine equipped with an eddy current dynamometer (Techno Mech, 4 S, 1-cylinder). Experiments were conducted at different loads (25–100%) with rated speeds of 1500 rpm, 220 bar injection pressure, and 23˚bTDC. The tests and data storage are done using the sensors and computerized data acquisition system. Experimental setup, engine specifications, and equipment details are shown in Fig. 3, and Table 5 respectively.

**Table 5:** Technical specifications of the test engine parameter specification.

Brand	Kirloskar Engines
Configuration	Single Cylinder
Displacement	Four Stroke, Water Cooled
Bore	87.5 mm
Stroke	110 mm
Max speed	1500 rpm
Rated power	3.5 Kw
CR range	17.5:1
Injection angle	23˚bTDC
Injection pressure	220 bar
Loading system	Eddy current dynamo meter
Combustion Principle	Compression-Ignition
Cooling system	Water cooling

The engine is first warmed with conventional diesel before mahua biodiesel enters the cylinder. Following the experiment, the engine is changed to normal diesel and allowed to run until the mahua biodiesel is no longer visible in the fuel line. The superfluous clog is subsequently removed from the fuel line using the fuel injection pump, which is then used for the following run. The blended biodiesel is fed into the engine and run continuously to acquire precise data and maintain consistent temperatures. Using an eddy current dynamometer for load regulation and the data collecting system for cylinder pressure monitoring provides results stored on a computer. Diesel flow is monitored using a burette. The time spent for each attempt is recorded, and the engine flow can be adjusted based on the specifications. A data-gathering system collects data to analyze the engine’s combustion characteristics and operational efficiency. This system has a piezoelectric pressure transducer and an engine crankshaft angle indicator. The data shows the HRR and peak CP are calculated using the mean value of the pressure transducer connected to the data-gathering system during a 50-cycle period. A thermocouple monitors the water temperature at the intake and exit and determines the exhaust gas temperature, which is then recorded in a data-gathering system. The engine pressure is measured using a differential U-tube manometer determines the air flow rate. The fuel gravity is measured using a U-tube manometer (accuracy ± 2%). A 437 C-type Smoke Meter is used to monitor smoke emissions, and an AVL exhaust gas analyzer is used to measure CO, HC, and NOx.

### Test procedure

The present study focuses on the diesel engine’s performance (BTE, BSFC), combustion (CP, HRR) and emission characteristics (HC, CO, NOx, and smoke) throughout a wide load range at a constant 1500 rpm. Initially, the test engine ran in diesel mode to obtain a baseline reading. The fuel was subsequently converted to mahua biodiesel blends (M10, M20, and M30), and the optimal blend was determined using trial data. NP concentrations of 25, 50, and 75 ppm were then tested across a wide load range using the best biodiesel blend. Finally, the RSM technique was applied to determine the optimal NP content for an outperforming blend.

The following formula is used to compute the different performance and combustion parameters employed in the current investigation (Eqs. (1–4)).

1 $$Brake\;power=\frac{2\mathrm\pi{NT}}{60\times1000}(kW)$$

Torque (T) = W×R (N-m) Where, W – Nett load (kg), R – Arm length (mm), N – speed (rpm).

2 $$Brake\;thermal\;efficiency=\frac{BP}{mf\times CV}\times100(\%)$$

Where, BP – Brake power (kW), m_f_– mass of fuel consumption (kg/s).

CV – Calorific value of fuel (kJ/kg).

3 $$Brake\;specific\;fuel\;consumption\;(BSFC)=\frac{mf}{BP}(kg/kW-hr)$$

4 $$Heat\;release\;rate\;HRR=\frac Y{Y-1}\lbrack p\times\frac{dV}{d\theta}\rbrack+\frac Y{Y-1}\lbrack V\times\frac{dp}{d\theta}\rbrack$$

where 𝛶 **–** specific heat ratio.

$\frac{dV}{d\theta}$ – Change in volume per change in crank angle, $\frac{dp}{d\theta}$ - Change in pressure per change in crank angle. 

### Uncertainty analysis

Uncertainties occur because of performing the same tests repeatedly, choosing the right instruments, changes in the environment, human mistakes, instrument accuracy, and the way the device works. The uncertainty of observed parameters was determined using the Gaussian distribution technique, with a confidence level of + 2σ. Measurements were collected under the same operating circumstances to evaluate the level of variability. Table 6 indicates the measuring instrument’s uncertainty. The uncertainty of the observed value is calculated using the formula (ΔXi) = 2si/X̅i100. In Equ. (5). The degree of inaccuracy is expressed, and the root-mean-square approach offers acceptable error limits for the calculated parameters as shown in Table 7. Using these all parameter values, the total uncertainty of the entire experiment is calculated using Eq. (6) and is found to be 2.51%.

**Table 6:** Measurement accuracy and uncertainty data of the instruments.

Instrument	Measurement	Principle	Range	Accuracy	Uncertainty
Encoder	Speed	Inductive principle	0–5000 rpm	± 1.5 rpm	± 0.16
Monometer	Flow pressure	-	0–200 mm	± 2 mm	1
Temperature sensor	temperature	RTD	0–1750˚C	-	± 0.15
Dynamometer	Torque and power	Eddy current	0 -3.5 kW	± 1 kW	± 0.9
Fuel flow burette	Flow	Calorimetric technique	0-100 ml	± 1%	± 0.1
Piezoelectric transducer	Pressure measurement	Piezo-electric effect	0–200 bar	± 5%	± 1
AVL Di test analyser	NO_X_ HC CO	Chemiluminescence Analyzer FID NDIR	0-5000 ppm 0–10,000 ppm 0–10% v	± 10 ppm ± 10 ppm ± 0.01% v	± 2.13% ± 1.12% ± 2.14%
437 C-Smoke meter	Smoke	Blocking of filter paper	0-100%	± 1	± 2.2

**Table 7:** All engine parameters uncertainty values.

S.no	Engine parameters	Uncertainty
1	Load	± 1.2 N
2	Speed	± 1.5 rpm
3	BTE	± 1.52%
4	BSFC	± 0.52%
5	CO	± 0.05% vol
6	HC	± 0.05 g/kWh
7	Smoke	± 0.02% vol
8	NOx	± 0.07 g/kWh

5 $$\triangle R=\surd\left\{(\partial R/\partial X_1)\triangle X_1\right\}^2+({(\partial R/\partial X_2)\triangle X_2)}^2+......+({(\partial R/\partial X_n)\triangle Xn)}^2\}$$

6 $$\begin{array}{c}Total\;uncertainty\;\%\\=\;\surd{(Load)}^2+{(speed)}^2+{(BTE)}^2+{(BSFC)}^2+{(Smoke)}^2+{(NO_X)}^2+{(HC)}^2+{(CO)}^2\\=\;\surd\;{(1.2)}^2\;+\;{(1.5)}^2+\;{(1.52)}^2\;+\;{(0.52)}^2\;+\;\;{(0.02)}^2\;+\;{(0.07)}^2\;+\;{(0.05)}^2\;+\;{(0.05)}^2\\=\;2.51\;\%\end{array}$$

## Design of experiment

**Figure 4:**
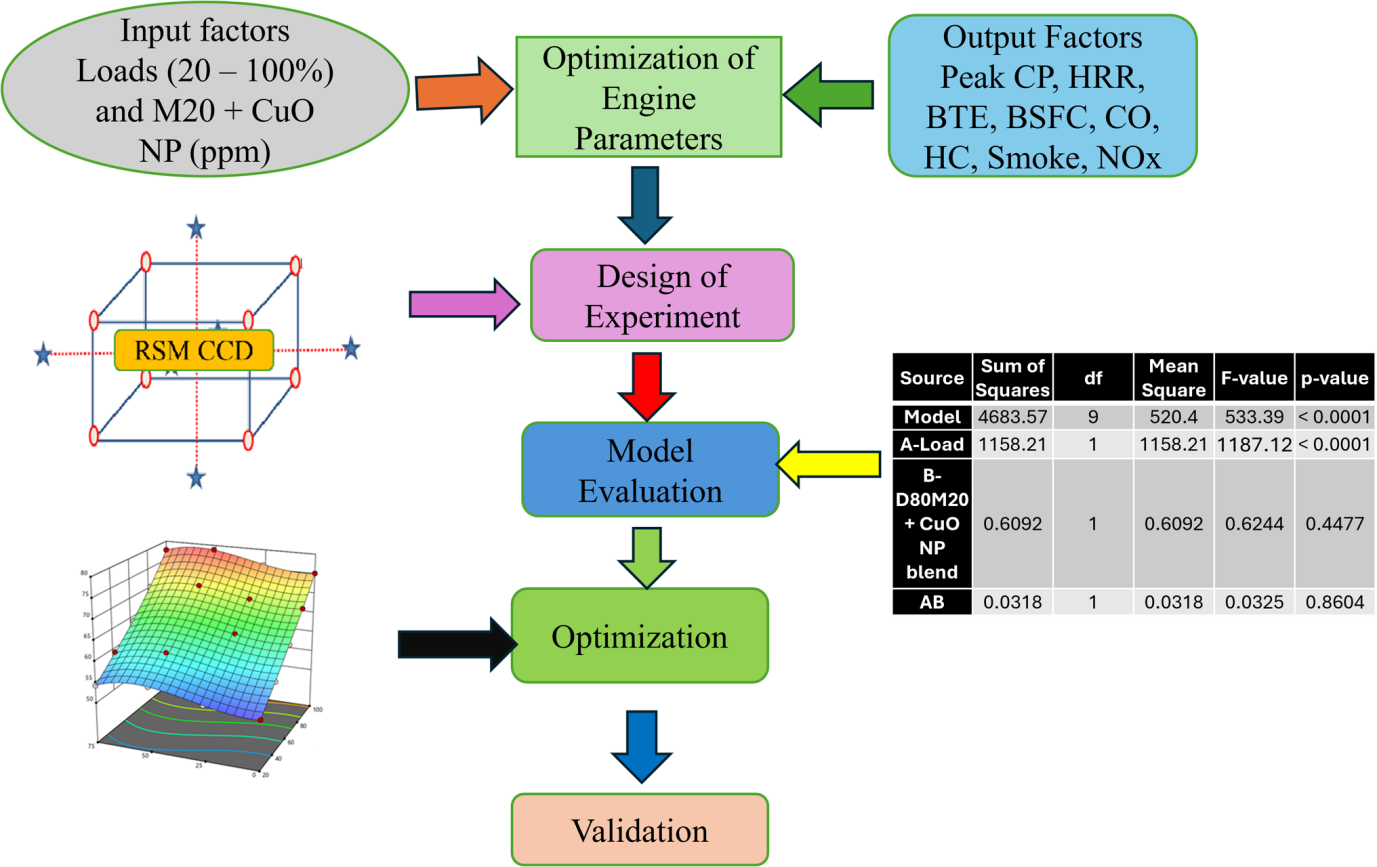
Central Composite Design model.

The design matrix responses in Table 8 were optimized using the CCD model (Fig. 4), and surface and contour plots were created for the parameters CP, HRR, BTE, BSFC, CO, HC, NOx, and Smoke. The regression models for each answer were created using a second-order regression equation, as shown in 5.1. Overall, the regression analysis utilizing the RSM reveals that the relationship between process and test parameters was as predicted. The Design Expert 13 software generates surface plots for two input variables while keeping the other variables constant as hold values. The hold values for all graphs were set to 20% biodiesel (% v) and CuO NP (25, 50, and 75 ppm) under different load conditions. These graphs aid in seeing the relationships between variables and their impacts on responses during the optimization process^29^.

**Table 8:** RSM design metrics.

Run	A: Load (%)	B: M20 + CuO NP (ppm)
1	20	0
2	20	25
3	20	50
4	20	75
5	40	0
6	40	25
7	40	50
8	40	75
9	60	0
10	60	25
11	60	50
12	60	75
13	80	0
14	80	25
15	80	50
16	80	75
17	100	0
18	100	25
19	100	50
20	100	75

### Regression equation in all parameters 

7 $$\begin{array}{c}CP\;(bar)\;=\;66.3387\;+\;20.9308\;\times\;A\;+\;2.36011\;\times\;B\;+\;0.366\\\times\;AB\;-\;6.10143\;\times\;A^2\;-\;0.189\;\times\;B^2+\;0.862286\;\times\;A^2B\\-\;0.65925\;\times\;AB^2\;-\;8.73833\;\times\;A^3\;-\;0.25425\;\times\;B^3\end{array}$$

8 $$\begin{array}{c}HRR\;(J/^\circ CA)=55.8134+35.0225\times A+0.879321\times B-0.0756\\\times AB-7.68571\times A^2-0.0585\times B^2+0.336857\times A^2B-0.603\\\times AB^2-16.69\times A^3+0.39825\times B^3\end{array}$$

9 $$\begin{array}{c}BTE\;(\%)\;=\;30.0003\;+\;8.89937\;\times\;A\;+\;0.493143\;\times\;B\\-\;0.2319\;\times\;AB\;-\;3.83357\;\times\;A^2\;-\;0.0405\;\times\;B^2\;-\;0.283286\\\times\;A^2B\;+\;0.014625\;\times\;AB^2\;-\;2.38\;\times\;A^3\;+\;0.6975\;\times\;B^3\end{array}$$

10 $$\begin{array}{c}BSFC\;(Kg/kW-hr)\;=\;0.344298\;-\;0.232494\;\times\;A\;-\;0.0219952\\\times\;B\;-\;0.006639\;\times\;AB\;+\;0.0480214\;\times\;A^2\;+\;0.00901125\;\times\;B^2\\+\;0.00481286\;\times\;A^2B\;+\;0.00502875\;\times\;AB^2\;+\;0.1279\;\times\;A^3\;-\;0.00271125B^3\end{array}$$

11 $$\begin{array}{c}CO\;(\%)\;=\;0.162074\;-\;0.0212646\;\times\;A\;-\;0.02008\;\times\;B\;+\;0.014703\\\times\;AB\;+\;0.0284929\;\times\;A^2\;-\;0.00117\;\times\;B^2\;+\;0.00303\;\times\;A^2B\;-\;0.0106313\\\times\;AB^2\;-\;0.0900167\;\times\;A^3\;+\;0.001035\;\times\;B^3\end{array}$$

12 $$\begin{array}{c}HC\;(ppm)\;=\;65.722\;-\;20.2567\;\times\;A\;-\;2.47863\;\times\;B\\-\;0.8034\;\times\;AB\;+\;3.29429\;\times\;A^2\;+\;1.58288\;\times\;B^2\;-\;0.108\\\times\;A^2B\;+\;0.0135\;\times\;AB^2\;+\;1.73667\;\times\;A^3\;-\;3.31538\;\times\;B^3\end{array}$$

13 $$\begin{array}{c}Smoke\;(\%)\;=\;44.6115\;-\;14.3656\;\times\;A\;-\;4.8667\;\times\;B\\-\;0.6309\;\times\;AB\;+\;5.28929\;\times\;A^2\;+\;1.61888\;\times\;B^2\;+\;1.49014\\\times\;A^2B\;-\;0.120375\;\times\;AB^2\;-\;3.78\;\times\;A^3\;-\;1.67738\;\times\;B^3\end{array}$$

14 $$\begin{array}{c}NOx\;(ppm)\;=\;874.321\;+\;197.5\;\times\;A\;-\;33.0279\;\times\;B\\-\;8.1024\;\times\;AB\;-\;25.0286\;\times\;A^2\;+\;22.3594\;\times\;B^2\;+\;6.228\\\times\;A^2B\;+\;10.737\;\times\;AB^2\;-\;51.42\;\times\;A^3\;-\;16.5701\;\times\;B^3\end{array}$$

### Analysis of model

ANOVA was used to analyze the model’s outputs and determine the statistical significance of the selected input parameters in relation to the preset constraints. Supplementary Table 1 represents the ANOVA results in CI engine parameters. The software indicates that these data were derived using a linear regression model. Process parameters with a p-value of less than 0.05 at the 95% confidence level are considered significant factors for the desired response characteristics. Low P-values have a greater impact on the linear model. The p-value for lack of fit is less than 0.05, suggesting that the assumed second-order model fits well. The ANOVA Table 9 presents the coefficients of determination R2, adj R2, and projected R2 for performance and emission response. A variance of less than 20% between the “pre R2” and “adj R2” values suggests that they are in satisfactory agreement. The MAPE values are calculated for the optimal levels to assess the precision of the estimated results^40^.

**Table 9:** Model evaluation.

	Peak CP (bar)	HRR (J/˚CA)	BTE (%)	BSFC (kg/kW-hr)	Smoke (%)	NO (ppm)	HC (ppm)	CO (%)
Std. dev.	0.7918054	0.9877432	0.3480421	0.0258320	1.0987691	3.1098762	1.3805437	0.02795
Mean	63.18	51.94	28.06	0.3733	42.16	874.23	53.56	0.1757
C.V (%)	1.25	1.90	2.67	5.79	4.20	0.3546	2.01	15.86
R^2^	0.9998	0.9995	0.9989	0.9899	0.9993	0.9990	0.9976	0.9799
Adjusted R^2^	0.9997	0.9991	0.9978	0.9818	0.9986	0.9981	0.9955	0.9999
Predicted R^2^	0.9990	0.9954	0.9936	0.9697	0.9952	0.9931	0.9905	0.9993
AP	357.0457	185.6059	132.0654	42.5542	87.7551	282.7744	69.9716	56.7170

## Results and discussion

### Combustion analysis - in-cylinder pressure

**Figure 5:**
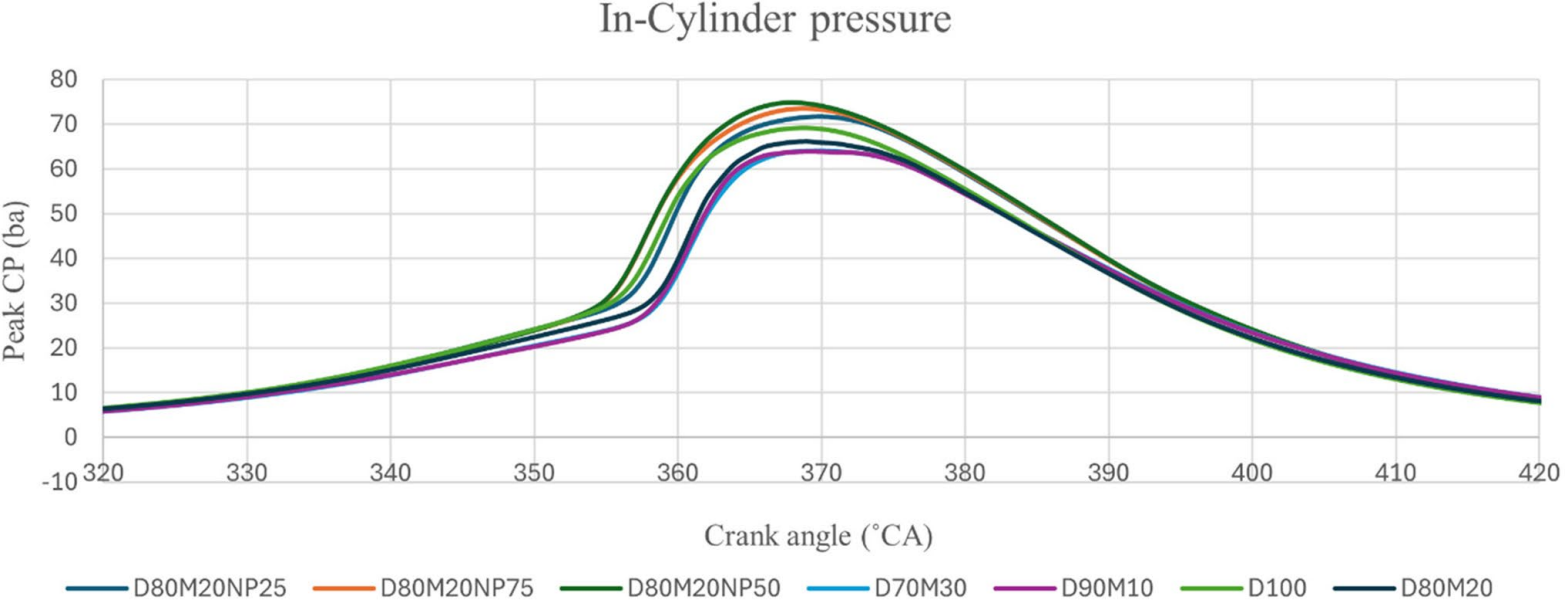
The effect of mahua and NP concentration on Peak CP.

CP is the pressure produced by burning air-fuel in the engine cylinder at the end of compression and the start of expansion. The ID period and injection geometry regulate fuel flow during rapid combustion, determining a diesel engine’s maximum CP. Fuel burning, O_2_ levels, viscosity, latent heat of vaporization, and CN are all factors that impact CP. Figure 5 observed peak CP values were 73.44 bar, 70.36 bar, 71.94 bar, 71.46 bar, 73.63 bar, 75.28 bar, and 74.62 bar for D100, M10, M20, M30, M20NP25, M20NP50, and M20NP75 respectively. The peak CP values of diesel and M20 blend are 72.24 bar and 71.94 bar, respectively. The M20 blend has a lower CP due to its increased density and viscosity. These fuel characteristics lead to a very poor evaporation rate and poor combustion.

**Figure 6:**
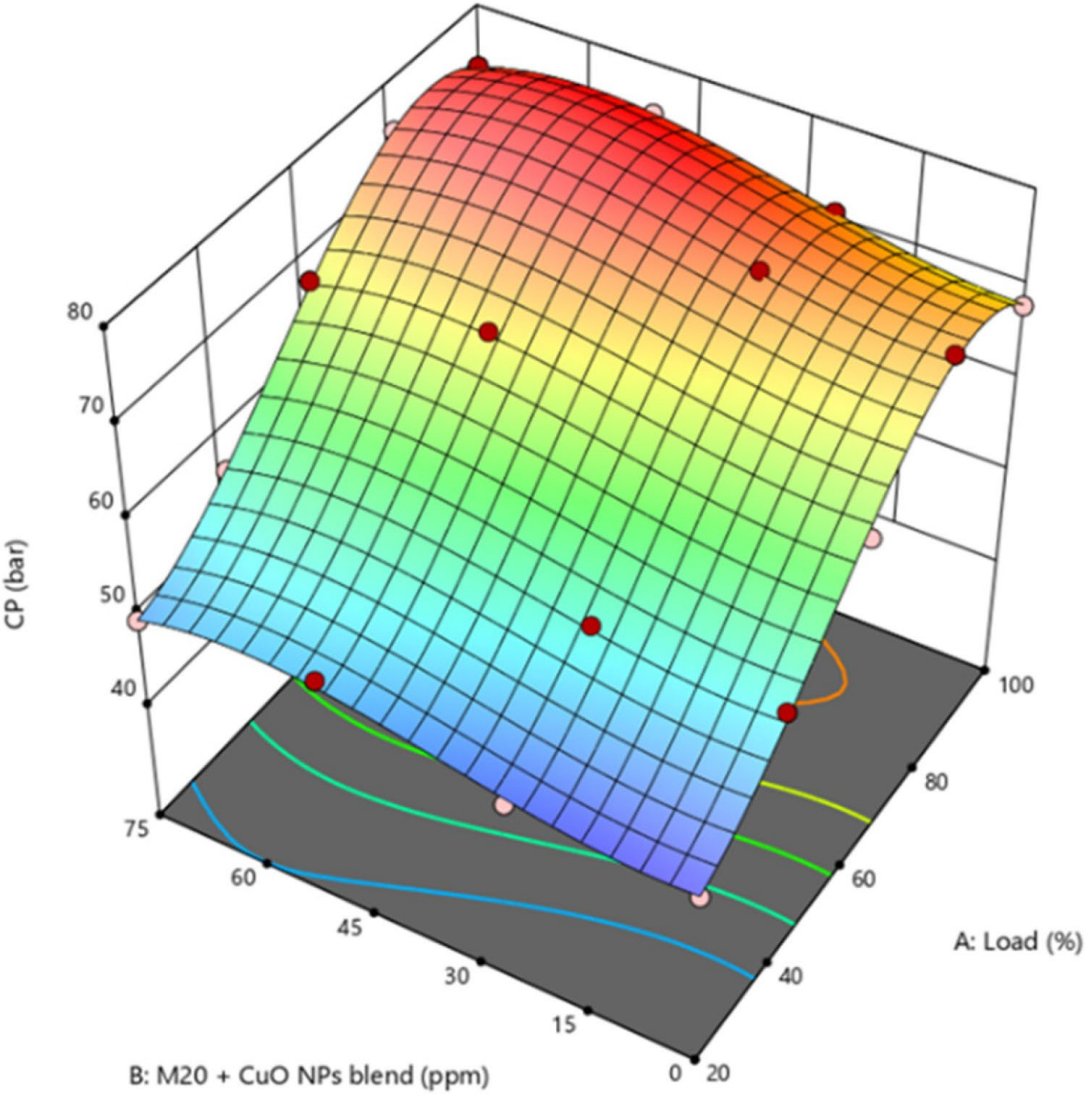
Impact of engine load and NP concentration on peak CP of M20 blend.

Therefore, there was a higher presence of unburned particles than D100^41^. The M20NP50 blend resulted in 4.6% greater peak CP than M20 and 2.5% greater than D100. M20 blend with CuO NP added fuel blends shows greater peak CP than M20 as the presence of CuO NP increases the fuel’s combustion rate by increasing its surface area and influences the ID time, resulting in enhanced peak CP and overall combustion performance^42^. It is shown in Fig. 6 that peak CP improves significantly when the M20 blend with CuO NP. The enhanced surface area of CuO NP and the excess of O_2_ in the biodiesel accelerate the oxidation of the biodiesel blend. This rise in peak CP caused the occurrence of several problems. Moreover, mixing CuO NP with biodiesel blends significantly increased CP more than diesel and biodiesel blends. A superior air-fuel (A/F) combination also increases cylinder fuel combustion rate.

### Heat release rate

**Figure 7:**
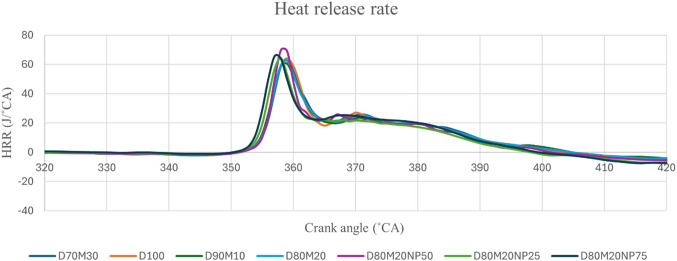
The effect of mahua and NP concentration on HRR.

The HRR analysis thoroughly summarises the various combustion phases at different engine intervals. HRR needs to investigate several kinds of parameters, including ID, the amount of burnt fuel at various phases of combustion, and the beginning and ending of combustion. Figure 7 demonstrate the HRR values were 64.31, 61.62, 62.23, 61.91, 63.70, 65.02, and 64.57 (J/deg) for D100, M10, M20, M30, M20NP25, M20NP50, and M20NP75 respectively. The HRR values of the diesel and M20 blend are 64.31 (J/˚CA) and 62.23 (J/˚CA), respectively.

**Figure 8:**
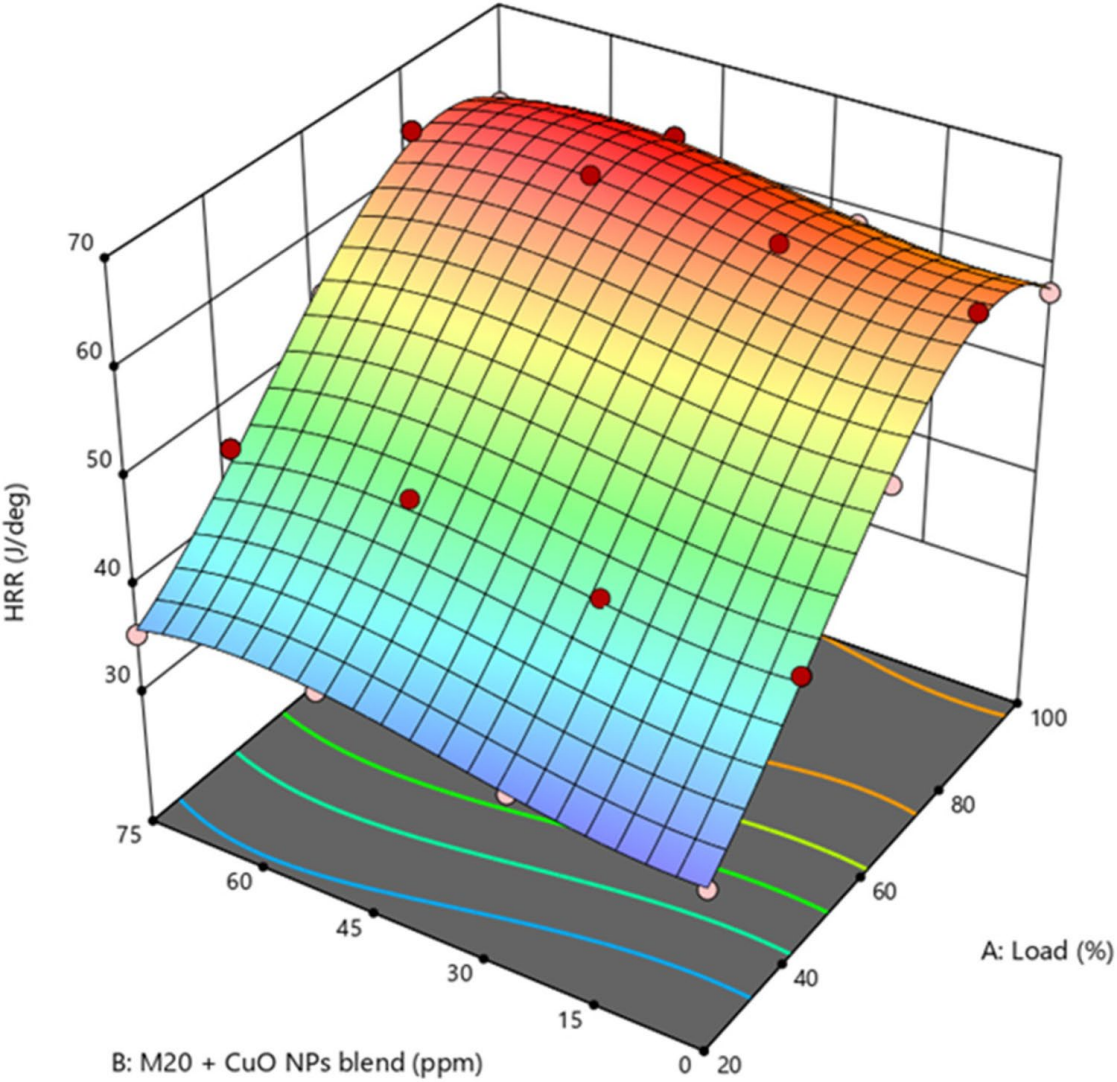
Impact of engine load and NP concentration on HRR of M20 blend.

M20 has a lower CV, greater viscosity, and lower volatility than diesel, which reduces the rate of HRR^43^. Figure 8 shows the M20NP50 blend resulted in 4.69% greater HRR than M20 and 1.1% higher than D100. The higher HRR of biodiesel fuels blended with nanoparticles may be due to the greater SA/V ratio of the nanoparticles, the increased CN, and lower ID, which leads to a higher evaporation rate and improved igniting properties of the fuel blends. Because the fuel accumulates more quickly during compression, the HRR is at its highest during the combustion phase involving premixed fuel blends^44^.

## Performance analysis

### Brake thermal efficiency

**Figure 9:**
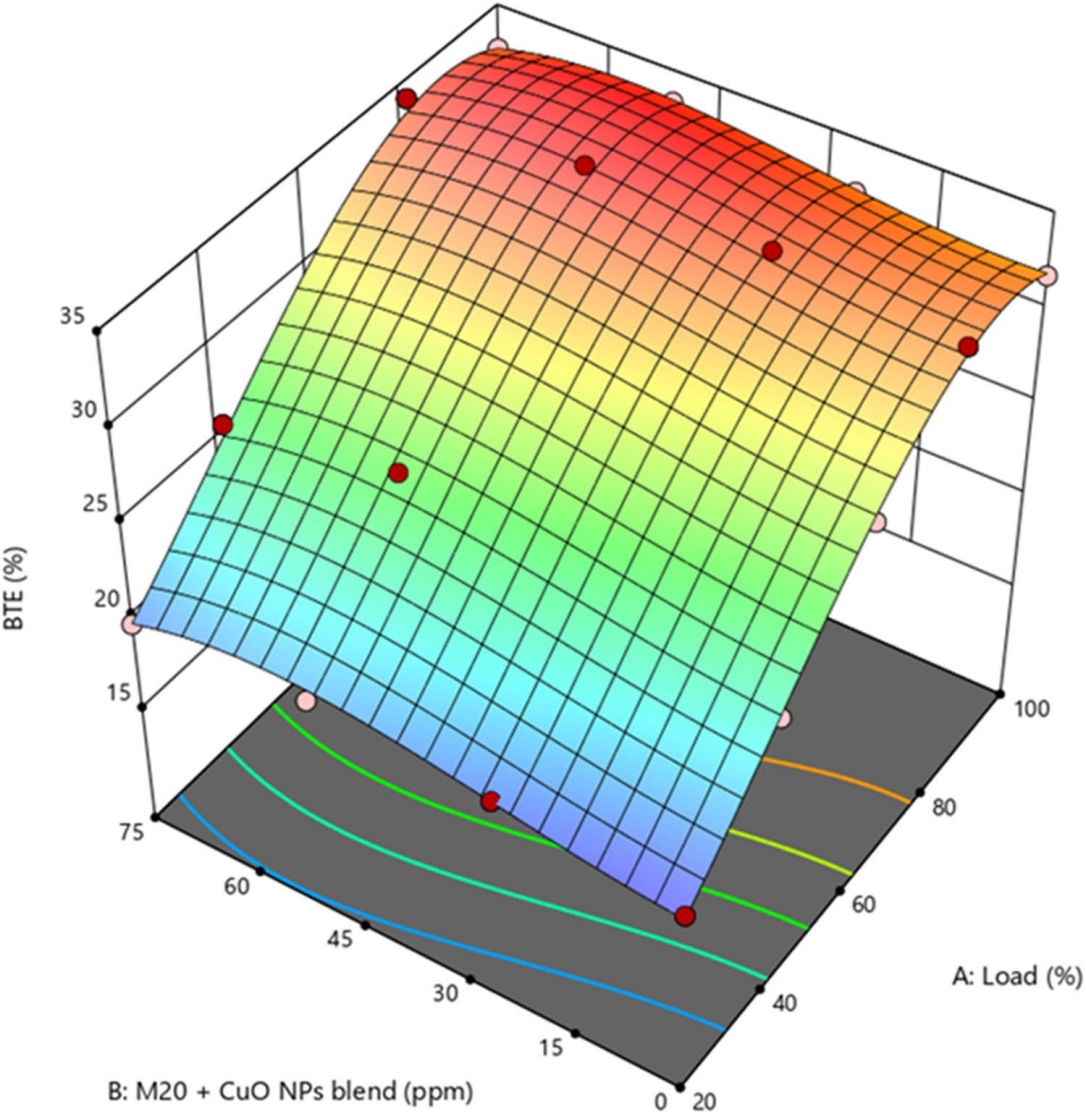
Impact of engine load and NP concentration on BTE of M20 blend.

BTE is an engine parameter that evaluates the efficiency of input fuel energy converted to mechanical output. Across all blended fuels, the BTE rises gradually as the load increases. Certain biodiesel characteristics, like viscosity, density, CV, and lubricity, significantly impact BTE and BP. Supplementary Fig. 1 illustrate the BTE values were 33.41%, 32.06%, 32.65%, and 32.45% for D100, M10, M20, and M30, respectively. M20 blend resulted in 2.27% lower BTE than D100. M20 blend has a lower BTE than diesel fuel due to the biodiesel blend’s lowered CV, greater viscosity, and density. Biodiesel’s high viscosity makes it atomize with bigger fuel droplets, forming a secondary A/F mixture. The increased viscosity of biodiesel blends leads to incomplete fuel breakdown inside the combustion chamber, resulting in a lower BTE^45^. Due to its superior performance, the M20 blend was selected as the optimal fuel blend for further experimental evaluation. Figure 9 represent the BTE values of M20 blended with CuO NP are M20NP25, M20NP50, and M20NP75 was found to be 33.55%, 34.15%, and 34.02%, respectively. M20NP50 showed 4.6% greater BTE than M20 and 2.2% greater than D100. Notably, adding CuO NP to M20 blend resulted in a significant increase in BTE. The dispersion of CuO NP leads to a higher CV of the fuel blend. Furthermore, the higher thermal conductivity of CuO NP leads to better combustion properties and heat transfer, improving BTE^46^. Supplementary Fig. 2 represents the actual vs. predicted values of BTE.

### Brake-specific fuel consumption

BSFC refers to the ratio of fuel consumed to BP. Researchers often favor lower values for BSFC since they indicate the engine’s efficiency in converting input energy into work output. It has been noted that BSFC tends to decrease as engine loads increase. Supplementary Fig. 3 observed the BSFC values were 0.2512, 0.2851, 0.2716, and 0.2791 kg/kW-hr for D100, M10, M20, and M30, respectively. M20 blend resulted in higher BSFC than diesel. The BSFC value is raised by M20 blend due to greater viscosity and lower CV^47^.

**Figure 10:**
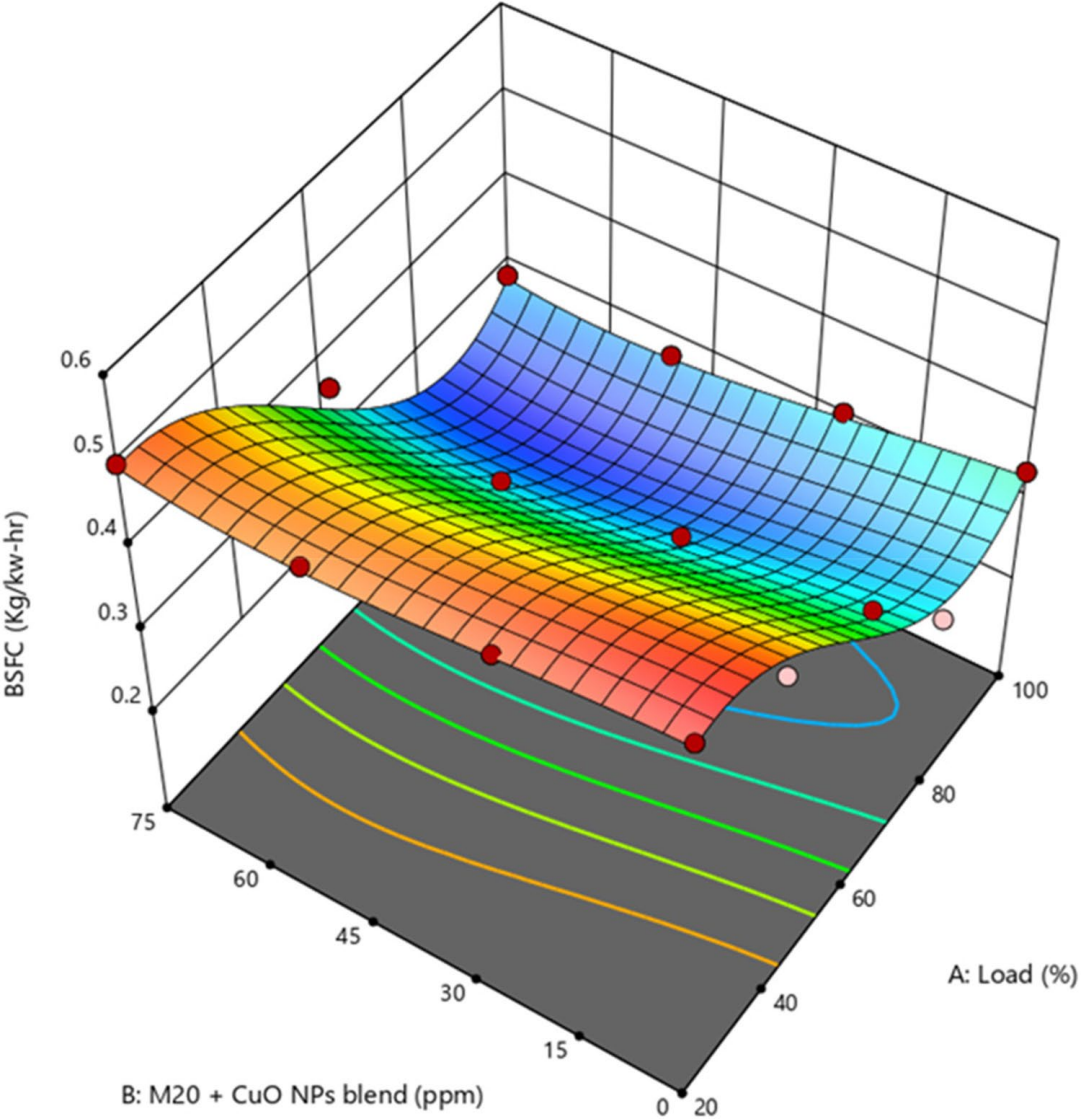
Impact of engine load and NP concentration on BSFC of M20 blend.

Based on superior performance, M20 was chosen as an optimum fuel blend for further experimental investigation. BSFC of the NP blends of M20NP25, M20NP50, and M20NP75 was found to be 0.2415, 0.2289, and 0.2314 kg/kW-hr, respectively. M20NP50 showed 9.7% less BSFC than D100. The M20NP50 and M20NP75 blends exhibited improved combustion, leading to a reduction in BSFC across all loads as shown in Fig. 10. A potential reason is that the combustion process was enhanced, and BSFC was decreased due to CuO blending, which increased the SA/V ratio and reduced the ID. According to these findings, the CuO NP enhanced atomization and improved combustion. The higher fuel quality resulted in a lower BSFC for adding NP to the M20 blend during synthesis compared to the M20 blend^48,49^. Supplementary Fig. 4 shows the actual vs. predicted values of BSFC.

## Emission analysis

### Carbon monoxide

**Figure 11:**
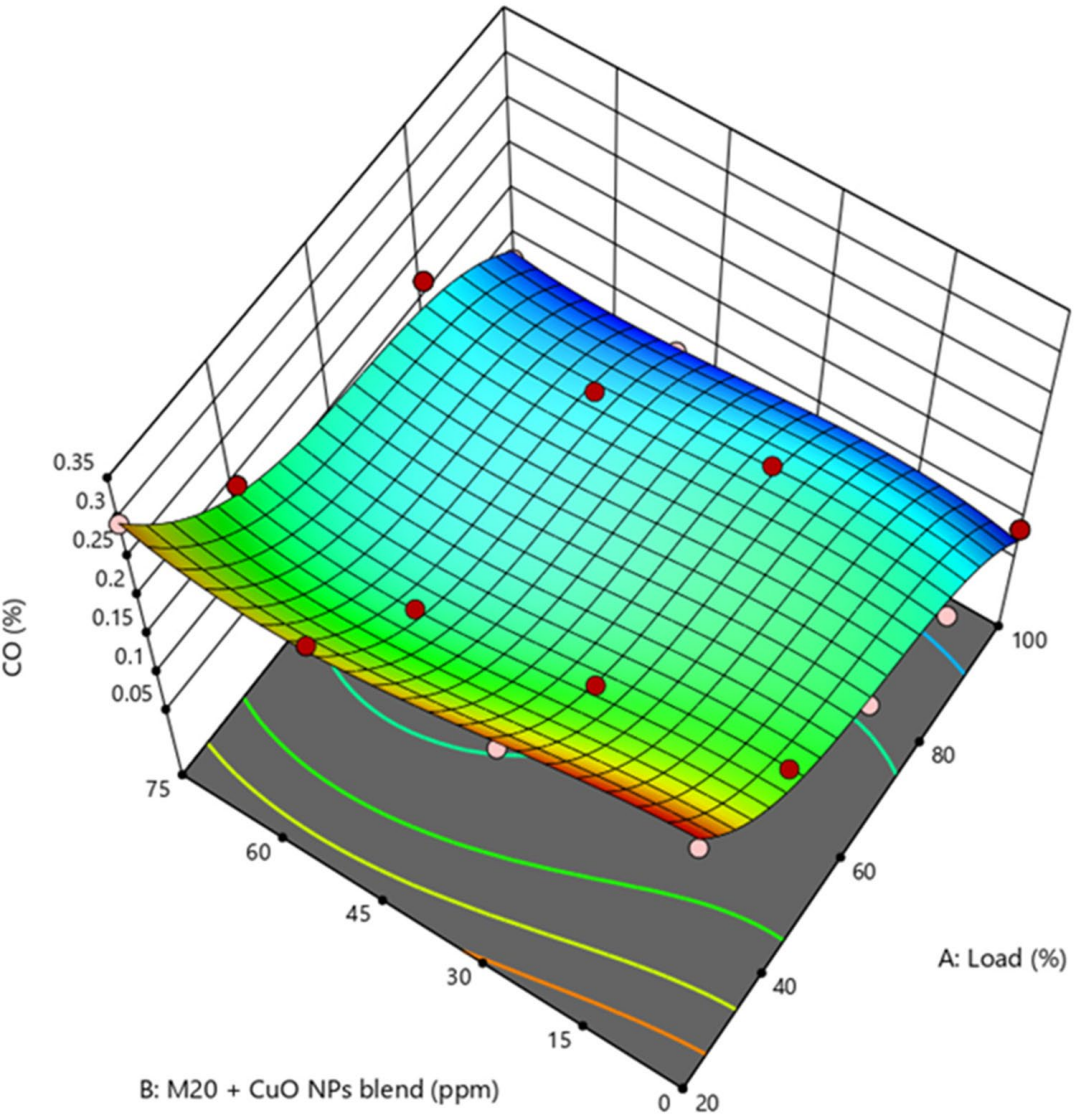
Impact of engine load and NP concentration on CO emission of M20 blend.

CO is a significant contributor to environmental pollution and adversely affects human health. Hence, it is important to examine and assess various aspects of CO. Higher CO. the higher A/F ratio at full load conditions leads to reduced CO oxidation as shown in supplementary Fig. 5. Diesel, M10, M20, and M30 exhibited CO levels of 0.0755, 0.0989, 0.0862, and 0.0937%, respectively. The biodiesel blends resulted in 30.9%, 14.17%, and 24.1% higher CO than diesel. because of higher viscosity, which promotes poor A/F mixing and atomization, leads to a lowering in the amount of CO_2_ conversion^50^. Figure 11 demonstrate the increased CO levels due to higher load conditions. The findings demonstrated that compared to the M20 blend, fuels containing CuO NP produced lower CO. M20NP50 blend resulted in 9.13% and 25.6% lower CO than the D100 and M20 blend. Adding CuO NP may increase the amount of fuel-air blending for uniform burning, resulting in complete combustion due to the reduced ID caused by CuO-blended fuels. Accordingly, when compared with the M20 blend, the CuO-blended fuels produce lower CO^51^. Supplementary Fig. 6 illustrate the actual vs. predicted values of CO.

### Hydrocarbon emission

**Figure 12:**
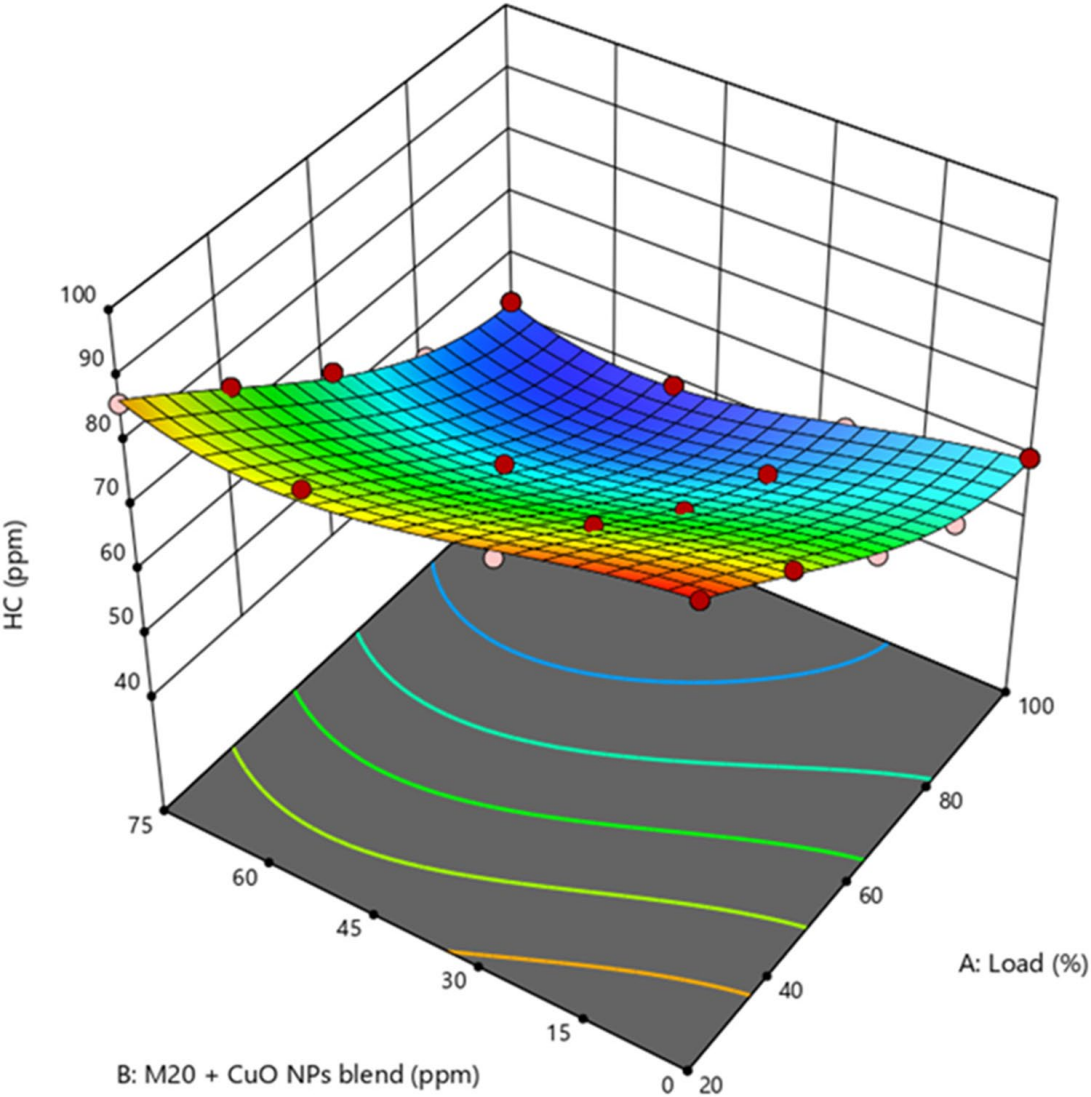
Impact of engine load and NP concentration on HC emission of M20 blend.

The study of HC reveals that fuel molecules were not burned completely. Because of incomplete combustion, unreacted HC exhaust degrades engine performance. Incomplete combustion is caused by insufficient air for burning. Supplementary Fig. 7 represents the HC emission levels were 52.54, 66.61, 60.14, and 63.46 ppm for Diesel, M10, M20, and M30 at higher loads. The HC emission of biodiesel blends rises by 19.5%, 7.9%, and 13.8% than diesel. The high viscosity of M20 blend increased the quench layer and deterioration of fuel atomization, and vaporization led to incomplete mixing and combustion. The engine load conditions and A/F ratio both influence HC. Excessive HC production occurred due to an increase in the quench zone and fuel deposition in cracks caused by more fuel injection into the combustion chamber at full load^52^. Figure 12 demonstrates the how concentration of CuO NP and engine load influence HC emissions. M20NP50 blend resulted in 6.8% and 22.2% lower HC than D100 and M20 blend. Adding CuO NP, which increases the amount of O_2_ accessible for combustion and accelerates combustion, improves the A/F mixture in the fuel injection, and NP propagation results in full combustion in the combustion chamber, resulting in lower HC emissions from the exhaust. The M20 blend generated more HC than NP-added fuel blends. Increasing the amount of NP in the fuel decreases the ID, resulting in longer premixed combustion phases and less HC formation^53^. Supplementary Fig. 8 shows the actual vs. predicted values of HC.

### Smoke emission

Smoke content in flue gases shows the presence of pollutants. It has been found that the transparency of smoke in flue gas increases with the load of each kind of fuel. Increasing engine load results in greater smoke emissions as shown in supplementary Fig. 9. Diesel, M10, M20, and M30 showed smoke values of 35.64, 44.76, 39.11, and 42.83%, respectively. The smoke from biodiesel blends increased by 25.5%, 9.7%, and 20.17% compared to diesel.

**Figure 13:**
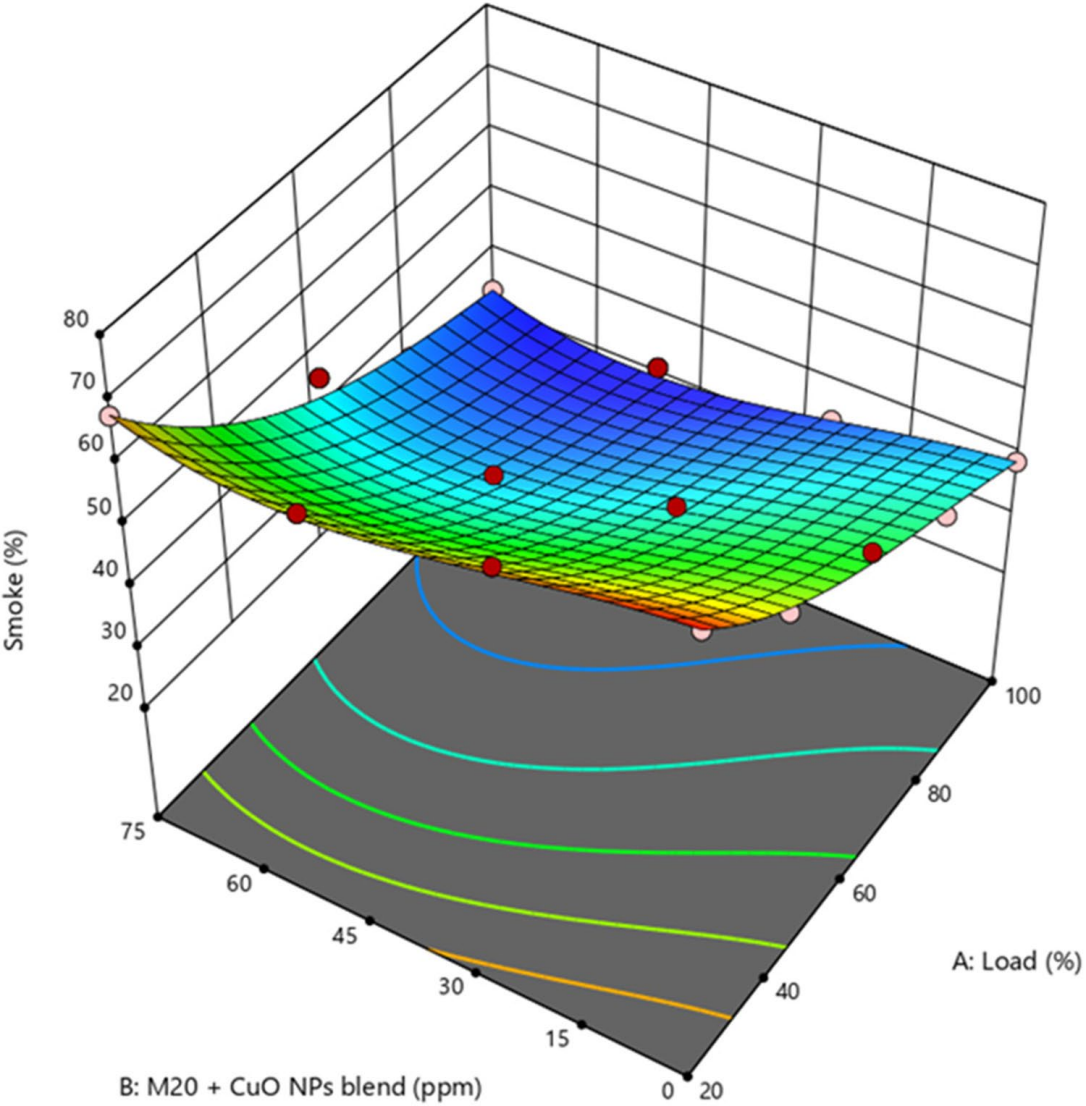
Impact of engine load and NP concentration on smoke emission of M20 blend.

The increasing smoke of biodiesel blends caused by increased viscosity and low penetration affects the atomization properties leading to poor combustion when compared to diesel^54^. Figure 13 illustrate the M20NP50 blend resulted in 12.1% and 19.9% lower smoke than the D100 and M20 blends. The shorter ID and improved ignition characteristics of the CuO NP-added fuel blends caused the drop in smoke. The blend of sulphur and O_2_ components largely caused the low smoke level. The reduced ID associated with the CuO NP-added fuel blends ensured enough fuel was supplied to the combustion chamber, producing complete combustion and minimum smoke emission^48^. Supplementary Fig. 10 represents the actual vs. predicted values of smoke.

### Nitrous oxide emission

NOx is produced when air nitrogen and O_2_ react at temperatures over 1500^◦^C near combustion. Supplementary Fig. 11 represents the differences in NOx emissions between the four test fuels. At full load, NOx emissions were obtained at 922.49, 832.63, 863.76, and 805.65 ppm for diesel, M10, M20, and M30. The NOx levels in biodiesel blends and diesel were reduced by 9.7%, 6.3%, and 12.7%, respectively. NOx levels were observed to drop as biodiesel content in the mixture increased. Reduced CV, higher biodiesel viscosity, and lower peak temperature might all help determine this effect due to poor A/F mixing and combustion^41^. Furthermore, adding CuO NP blend with M20NP25, M20NP50, and M20NP75 was found to be 917.87, 941.03, and 935.54 ppm, respectively. M20NP50 blend produced 8.9% more NOx than M20 without NP and 2% more than diesel.

**Figure 14:**
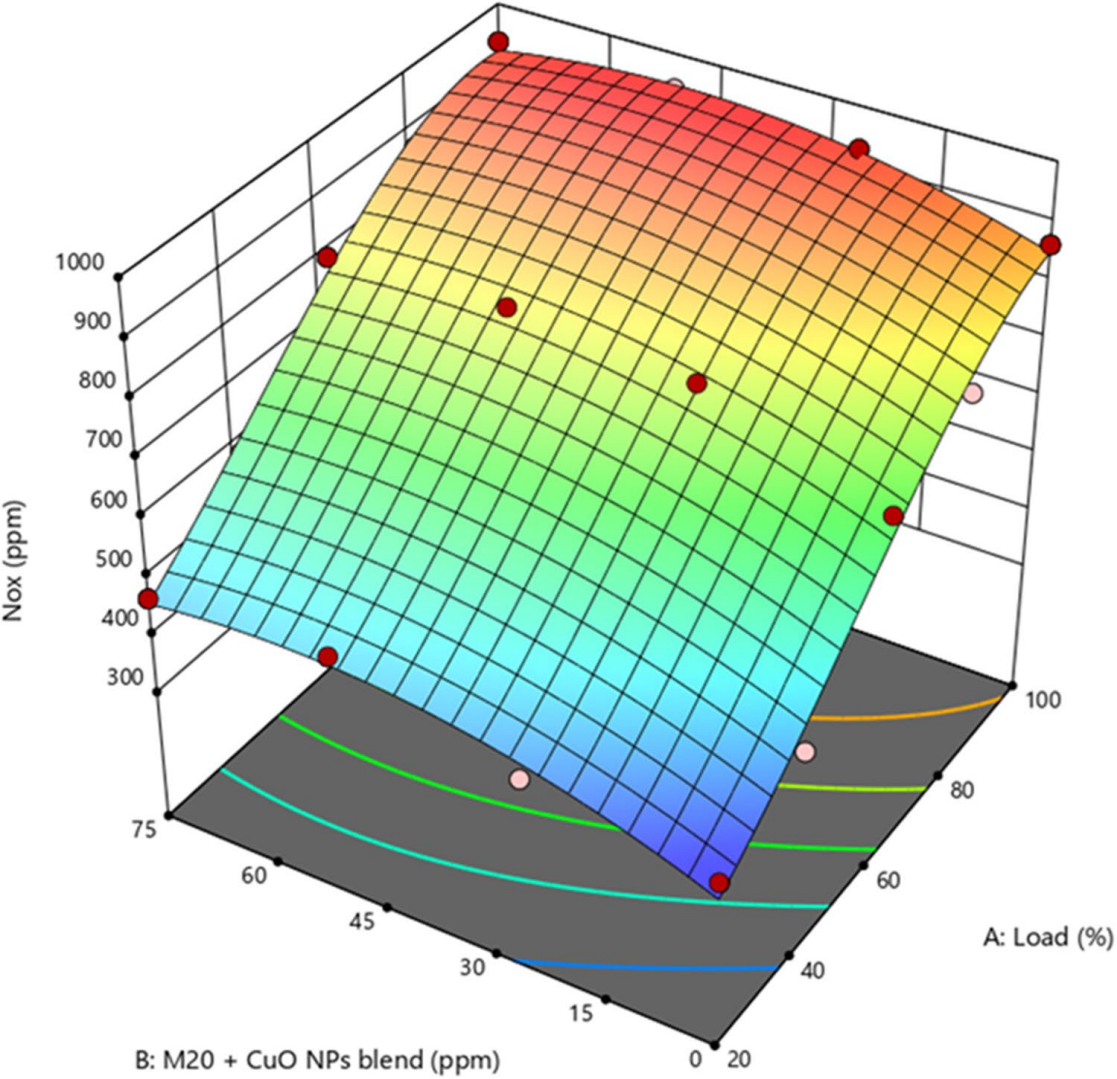
Impact of engine load and NP concentration on NOx emission of M20 blend.

Figure 14 demonstrate the NOx Changes trend as a function of load conditions and NP concentration in M20. It was found that the M20NP50 blend produced the highest levels of NOx. This might be due to complete combustion, which increases NOx emissions. It might be attributed to the growth of catalytic activity caused by NP, allowing for the use of additional O_2_ to improve combustion^55,56^. Supplementary Fig. 12 shows the actual vs. predicted values of NOx.

#### Optimization

The optimisation focuses on supporting load and NP concentration in selecting the M20 blend. We applied weights to determine the desired value to meet the implementation requirements and achieve the highest value of BTE while keeping the BSFC as low as possible. A weight value of “0.1” is chosen as the default to modify the appearance of specific functions. By assigning a significance rating, we emphasized the importance of all goals. Table 10 presents the results of optimizing the input and response variables numerically. Optimising performance and emissions is achieved by considering the mixture of M20 blend with CuO NP concentration at 80% engine load as shown in Table 11, which has a high desirability value of 0.9740. Table 12 shows the level of desirability for all constraints in the optimal solution after the optimization procedure^57^. The results of the RSM were confirmed by conducting experiments. The RSM optimizer provided the identical set of input variables that were used in three successive engine trials. The RSM’s predicted values were compared to the average values from the engine testing. All of the data as shown in supplementary Table 2, including experimental, predicted, and error calculations, are within a reasonable range, proving that the RSM predictions are reliable.

**Table 10:** Engine Input Level.

Engine Input	Code	Level		
		-1	0	1
Load (%)	A	20 20 100		
M20 + CuO NP (ppm)	B	0 25 75		

**Table 11:** Experimental validation.

Parameters	Experimental outcomes	RSM prediction data	Error (%)	Mean absolute Percentage Error (MAPE)
Load (%)	80	77.86	2.65	**3.0962**
M20 + CuO NP (ppm)	60	58.32	2.8	
Peak CP (bar)	73.46	71.13	3.17	
HRR (J/˚CA)	60.32	61.91	3.28	
BTE (%)	32.71	33.32	1.86	
BSFC (kg/kW-hr)	0.2678	0.2771	3.47	
Smoke (%)	30.24	31.46	4.05	
NOx (ppm)	768.54	807.98	4.88	
HC (ppm)	48.12	50.02	3.95	
CO (%)	0.0672	0.0689	2.48	

**Table 12:** Constraints.

Name	Goal	Lower Limit	Upper Limit	Lower Weight	Upper Weight	Importance	Desirability
A: Load (%)	In range	20	100	1	1	3	1
B: M20 + CuO NP blend (ppm)	In range	0	75	1	1	3	1
CP (bar)	Maximise	45.78	76.18	1	1	3	0.9998
HRR (J/˚CA)	Maximise	62.23	65.02	1	1	3	0.9995
BTE (%)	Maximise	18.5	34.15	1	1	5	0.9989
BSFC (kg/kW-hr)	Minimise	0.2289	0.5202	1	1	3	0.9887
CO (%)	Minimise	0.0686	0.332	1	1	1	0.9799
HC (ppm)	Minimise	49.2	94.32	1	1	1	0.9993
Smoke (%)	Minimise	31.32	74.09	1	1	1	0.9990
Nox (ppm)	Minimise	805.65	941.03	1	1	5	0.9976

#### Limitations of using CuO as a catalyst

The increased viscosity and pressure drop are effects of the greater concentration of CuO NP blended with D80M20. The engine’s performance drops as the concentration of CuO NP increased beyond certain limit (60 ppm in this case).

### Conclusion

The experiments were conducted to determine the optimal blending of mahua biodiesel blends and CuO NP in CI mode at varying loads with a constant speed.

- The BTE values for diesel, M10, M20, and M30 were 33.41%, 32.06%, 32.65%, and 32.45%, respectively. After analyzing the performance data, M20 was selected for further investigation due to its moderate performance and reduced emission compared to other blends.
- Among different concentrations (25, 50, and 75 ppm) of CuO NP with M20, M20NP50 blend resulted in 34.15% higher BTE and 0.2289 kg/kw-hr lower BSFC. This improvement was 4.6% and 15.72% higher than the M20 blend without NP, and 2.2% and 8.8% higher than diesel respectively. HC, CO, and smoke emissions decreased by 22.2%, 25.6%, and 19.9%, respectively, compared to the M20 blend without NP and reduced by 6.89%, 9.13%, and 12.1%, compared to diesel respectively. However, NOx emissions increased by 8.9% compared to the M20 blend without NP and by 2% compared to diesel.
- The RSM findings showed that M20 blended with 60 ppm NP at 80% load had the highest desirability (0.9740), and the proposed model accurately predicted engine responses with a MAPE of 3.0962%. When comparing the M20NP60 blend with diesel, BSFC improved by 14.5%, but BTE decreased by 4.22%. HC, CO, NOx, and smoke emissions were decreased by 2.24%, 2.04%, 22.4%, and 16.6%, respectively, at 80% load condition.
- This paper validates the engine parameters using RSM optimization to improve the performance and emission characteristics.

## Supplementary Information

Below is the link to the electronic supplementary material.

Supplementary Material 1

## Data Availability

The datasets used and/or analysed during the current study available from the corresponding author on reasonable request.

## Nomenclature

BSFC: Brake-specific fuel consumption (kg/kW-hr)
BTE: Brake thermal efficiency (%)
BTDC: Before top dead centre (˚ CA)
CA: Crank angle (˚ CA)
CI: Compression ignition
CN: Cetane number
CP: Cylinder pressure (bar)
CV: Calorific value (kJ/kg)
CO: Carbon monoxide (%)
FFA: Free fatty acid
HRR: Heat release rate (J/˚CA)
HC: hydrocarbons (ppm)
ICE: Internal combustion engine
ID: Ignition delay
Kg/kWh: Kilogram per kilowatt-hour
kJ/kWh: Kilojoule per kilowatt-hour
NO_X_: Nitric oxide (ppm)
NP: Nano particle
Ppm: Parts per million
