# Supplementary material for: Investigation on CuO nanoparticle enhanced mahua biodiesel/diesel fuelled CI engine combustion for improved performance and emission abetted by response surface methodology
Source: Sci Rep. 2024 Nov 6;14:26882. doi: 10.1038/s41598-024-77271-3 (PMC11542028; doi:10.1038/s41598-024-77271-3)
Supplement: Supplementary file 1 — Supplementary Material 1 [file 41598_2024_77271_MOESM1_ESM.docx]

**Supplementary files**

**Supplementary table 1 - ANOVA Results for CI engine parameters**

|  | **Peak CP** |  | **HRR** |  | **BTE** |  | **BSFC** |  | **CO** |  | |
| --- | --- | --- | --- | --- | --- | --- | --- | --- | --- | --- | --- |
| **Source** | **F-value** | **p-value** | **F-value** | **p-value** | **F-value** | **p-value** | **F-value** | **p-value** | **F-value** | **p-value** | |
| **Model** | 354.61 | < 0.0001 | 533.39 | < 0.0001 | 107.20 | < 0.0001 | 29.21 | < 0.0001 | 17.55 | <0.0001 |  |
| A-Load | 659.84 | < 0.0001 | 1187.12 | < 0.0001 | 133.08 | < 0.0001 | 76.58 | < 0.0001 | 0.5503 | 0.4753 | |
| B-D80M20 + CuO NP blend | 7 | 0.0245 | 0.6244 | 0.4477 | 0.3410 | 0.5722 | 0.5719 | 0.4669 | 0.4094 | 0.5367 | |
| AB | 1.19 | 0.3015 | 0.0325 | 0.8604 | 0.5317 | 0.4826 | 0.3674 | 0.5579 | 1.55 | 0.2418 | |
| A² | 207.83 | < 0.0001 | 211.91 | < 0.0001 | 91.53 | < 0.0001 | 12.11 | 0.0059 | 3.66 | 0.0847 | |
| B² | 0.2251 | 0.6454 | 0.0139 | 0.9086 | 0.0115 | 0.9166 | 0.4813 | 0.5036 | 0.0070 | 0.9351 | |
| A²B | 2.31 | 0.1598 | 0.2261 | 0.6446 | 0.2777 | 0.6097 | 0.0676 | 0.8002 | 0.0230 | 0.8825 | |
| AB² | 1.37 | 0.2691 | 0.7362 | 0.411 | 0.0008 | 0.9787 | 0.0749 | 0.7898 | 0.2877 | 0.6034 | |
| A³ | 109.62 | < 0.0001 | 256.96 | < 0.0001 | 9.07 | 0.0131 | 22.09 | 0.0008 | 9.40 | 0.0119 | |
| B³ | 0.0815 | 0.7811 | 0.1284 | 0.7275 | 0.6841 | 0.4275 | 0.0087 | 0.9275 | 0.0011 | 0.9743 | |
|  |  |  |  |  |  |  |  |  |  |  | |

|  | **HC** |  | **Smoke** |  | **NOx** |  |  |  |  |  |
| --- | --- | --- | --- | --- | --- | --- | --- | --- | --- | --- |
| **Source** | **F-value** | **p-value** | **F-value** | **p-value** | **F-value** | **p-value** |  |  |  |  |
| **Model** | 230.90 | < 0.0001 | 97.90 | < 0.0001 | 3296.87 | < 0.0001 |  |  |  |  |
| A-Load | 204.89 | < 0.0001 | 47.72 | < 0.0001 | 3832.38 | < 0.0001 |  |  |  |  |
| B-D80M20 + CuO NP blend | 2.56 | 0.1407 | 4.57 | 0.0583 | 89.43 | < 0.0001 |  |  |  |  |
| AB | 1.90 | 0.1985 | 0.5415 | 0.4787 | 37.95 | 0.0001 |  |  |  |  |
| A² | 20.09 | 0.0012 | 23.98 | 0.0006 | 228.13 | < 0.0001 |  |  |  |  |
| B² | 5.23 | 0.0452 | 2.54 | 0.1424 | 205.51 | < 0.0001 |  |  |  |  |
| A²B | 0.0120 | 0.9150 | 1.06 | 0.3281 | 7.85 | 0.0188 |  |  |  |  |
| AB² | 0.0002 | 0.9893 | 0.0070 | 0.9349 | 23.69 | 0.0007 |  |  |  |  |
| A³ | 1.44 | 0.2585 | 3.15 | 0.1064 | 247.60 | < 0.0001 |  |  |  |  |
| B³ | 4.59 | 0.0577 | 0.5444 | 0.4776 | 22.57 | 0.0008 |  |  |  |  |
|  |  |  |  |  |  |  |  |  |  |  |

**Supplementary table 2 - RSM Experimental data**

| **Load (%)** | **D80M20 + CuO NP blend (ppm)** | **CP (bar)** | **HRR (J/˚CA)** | **BTE (%)** | **BSFC (Kg/kw-hr)** | **CO (%)** | **HC (ppm)** | **Smoke (%)** | **Nox (ppm)** |
| --- | --- | --- | --- | --- | --- | --- | --- | --- | --- |
| 20 | 0 | 45.78 | 28.77 | 18.5 | 0.5202 | 0.332 | 94.32 | 74.09 | 361.09 |
| 40 | 0 | 54.47 | 37.23 | 23.5 | 0.4623 | 0.254 | 84.21 | 60.71 | 420.87 |
| 60 | 0 | 62.49 | 54.37 | 28.52 | 0.4103 | 0.167 | 72.21 | 54.43 | 672.54 |
| 80 | 0 | 71.94 | 67.54 | 32.65 | 0.2716 | 0.1242 | 63.12 | 45.03 | 741.75 |
| 100 | 0 | 68.13 | 64.42 | 31.8 | 0.3337 | 0.0862 | 60.14 | 39.11 | 863.76 |
| 20 | 25 | 46.57 | 29.32 | 19.61 | 0.5031 | 0.3021 | 87.65 | 70.21 | 403.87 |
| 40 | 25 | 55.21 | 37.86 | 24.62 | 0.4411 | 0.2134 | 78.43 | 52.43 | 563.87 |
| 60 | 25 | 65.46 | 56.63 | 29.57 | 0.3889 | 0.1456 | 66.89 | 48.9 | 782.76 |
| 80 | 25 | 73.43 | 68.11 | 33.55 | 0.2415 | 0.1854 | 59.11 | 38.8 | 843.21 |
| 100 | 25 | 71.06 | 66.08 | 32.33 | 0.3051 | 0.0732 | 53.48 | 34.02 | 928.87 |
| 20 | 50 | 51.31 | 32.54 | 20.21 | 0.4913 | 0.2842 | 85.43 | 65.35 | 488.45 |
| 40 | 50 | 56.95 | 38.76 | 27.33 | 0.4263 | 0.1702 | 70.32 | 48.59 | 601.43 |
| 60 | 50 | 68.91 | 58.5 | 30.39 | 0.3505 | 0.1189 | 62.31 | 41.61 | 805.98 |
| 80 | 50 | 76.18 | 69.42 | 34.15 | 0.2289 | 0.1537 | 51.96 | 31.72 | 878.51 |
| 100 | 50 | 74.83 | 67.57 | 33.38 | 0.2781 | 0.0686 | 49.2 | 31.31 | 941.03 |
| 20 | 75 | 49.42 | 30.38 | 19.73 | 0.501 | 0.2954 | 86.33 | 67.83 | 467.41 |
| 40 | 75 | 56.02 | 38.08 | 25.67 | 0.4346 | 0.1948 | 75.98 | 50.64 | 581.87 |
| 60 | 75 | 67.21 | 57.34 | 28.93 | 0.3621 | 0.1259 | 65.58 | 45.68 | 791.43 |
| 80 | 75 | 74.62 | 68.92 | 34.02 | 0.2314 | 0.1704 | 55.49 | 35.31 | 863.21 |
| 100 | 75 | 73.67 | 66.92 | 32.76 | 0.2843 | 0.0702 | 52.51 | 33.42 | 935.54 |

**Supplementary figures:**


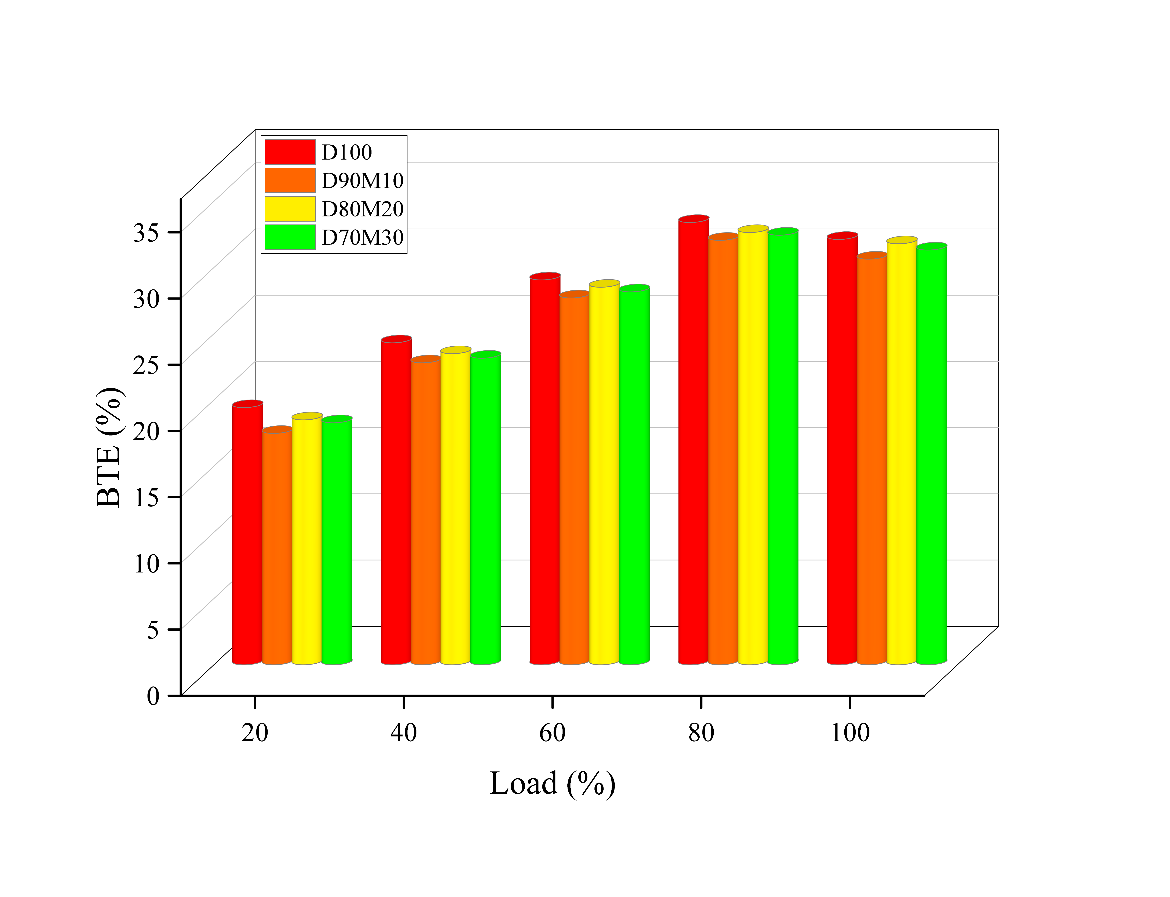


Supplementary fig 1. Impact of engine load and mahua biodiesel blend ratio on BTE


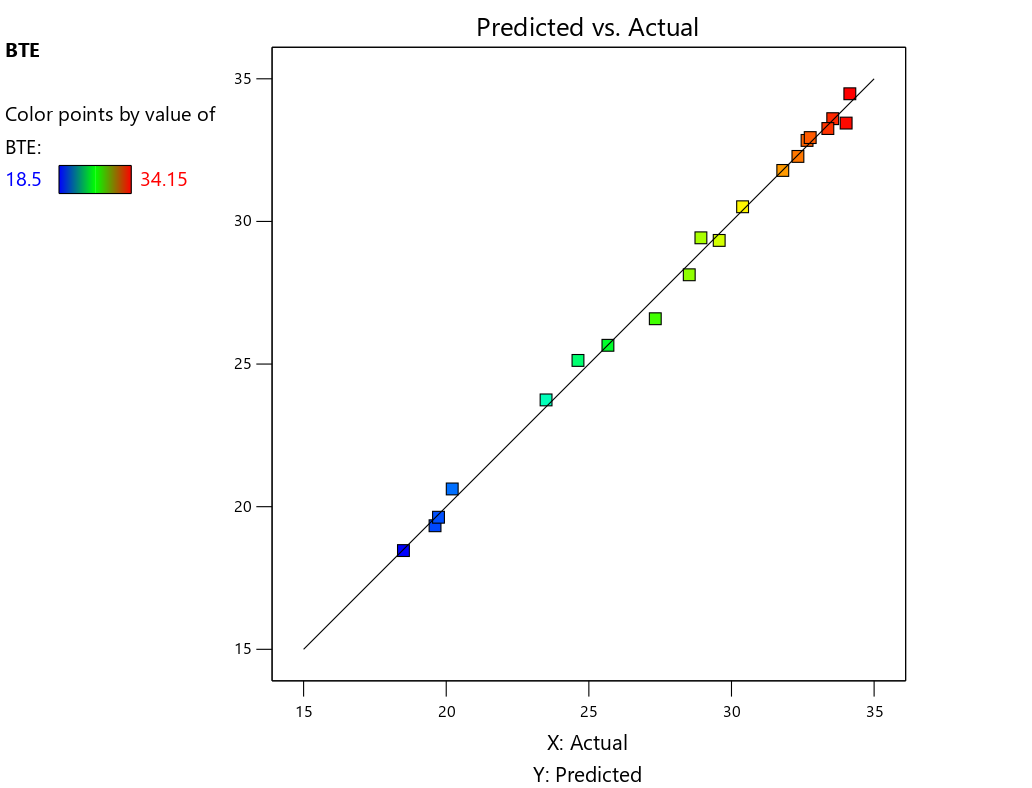


Supplementary fig 2. Actual vs predicted values of BTE


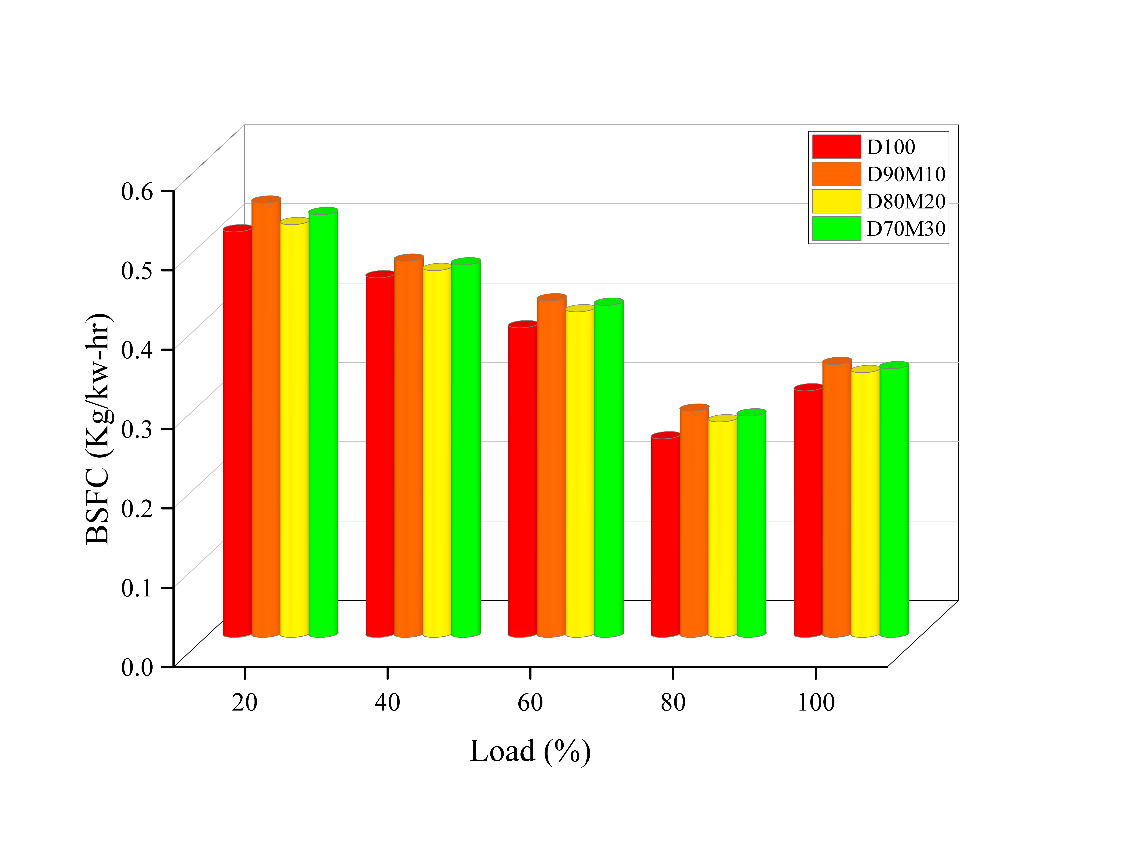


Supplementary fig 3. Impact of engine load and mahua biodiesel blend ratio on BSFC


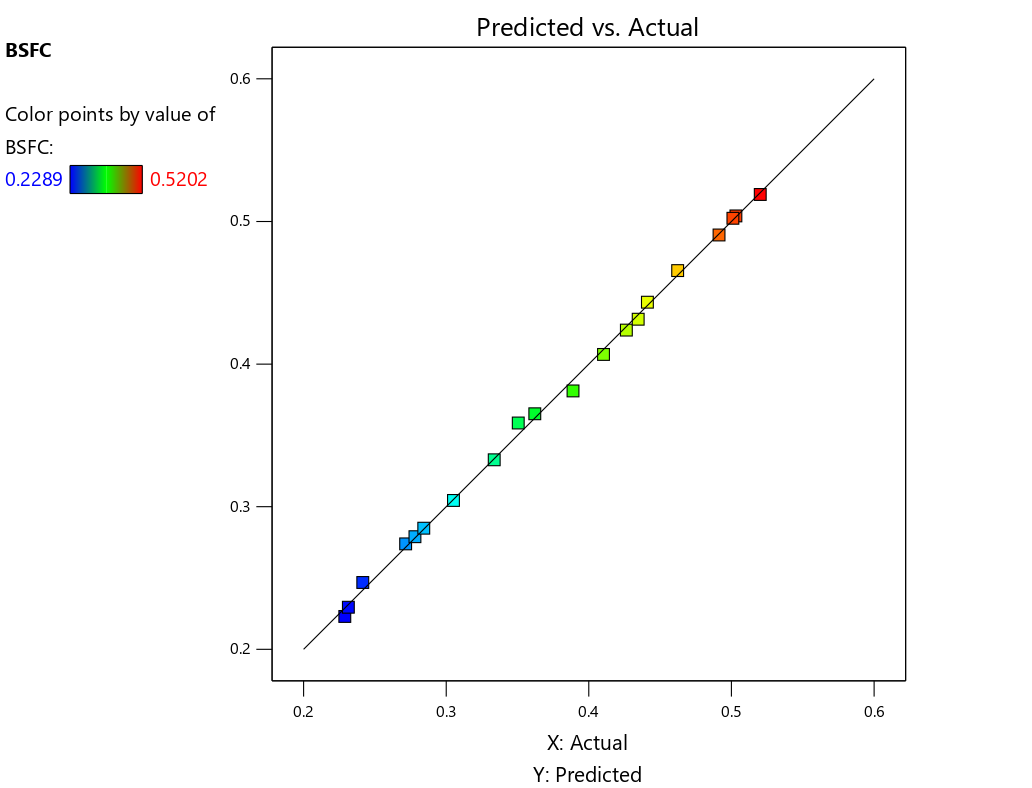


Supplementary fig 4. Actual vs predicted values of BSFC


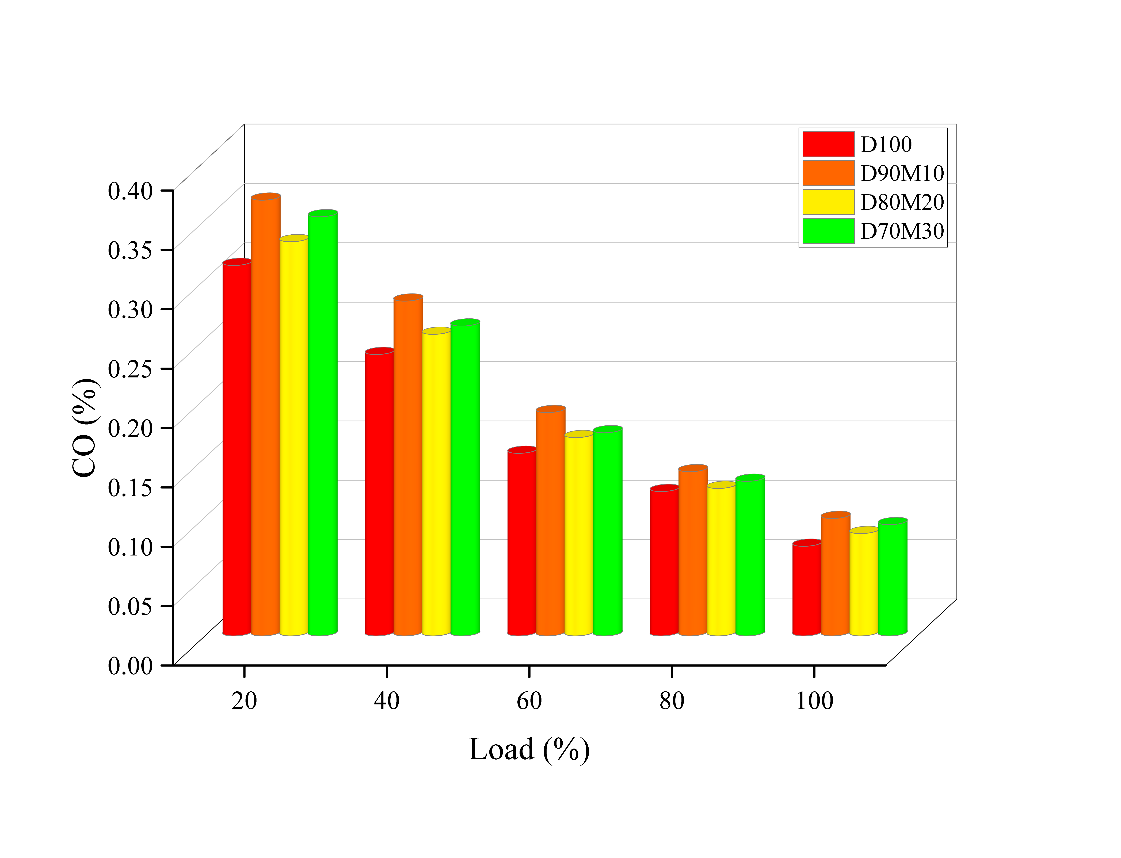


Supplementary fig 5. Impact of engine load and mahua biodiesel blend ratio on CO emission


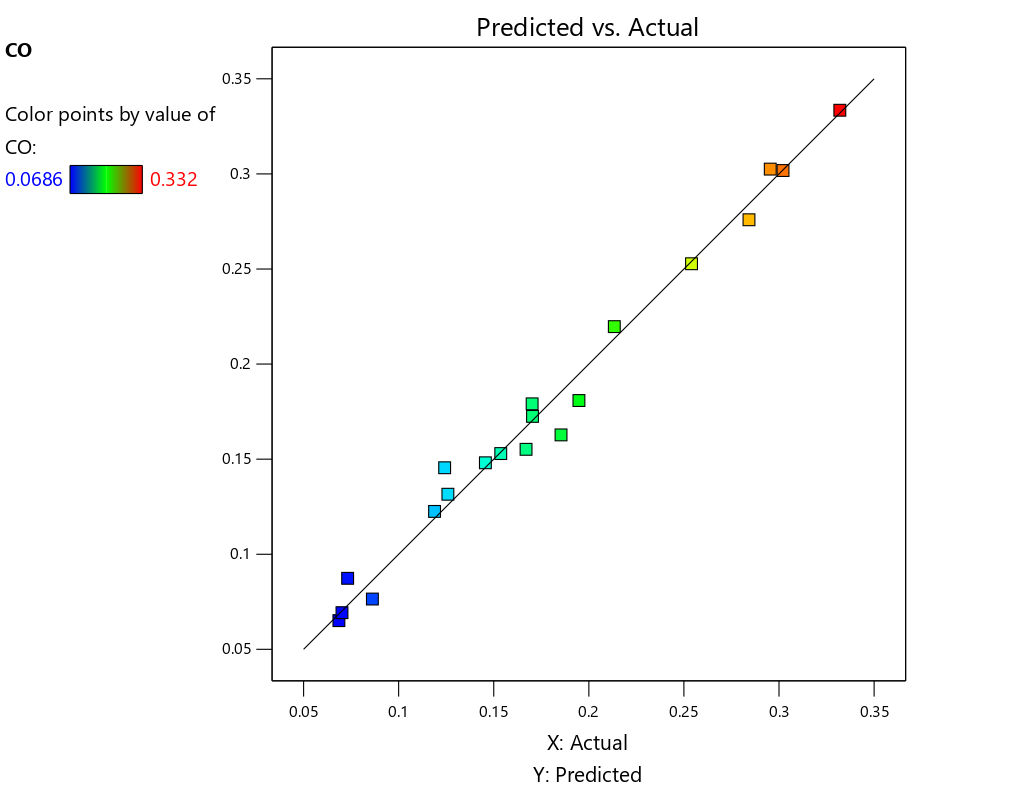


Supplementary fig 6. Actual vs predicted values of CO emission


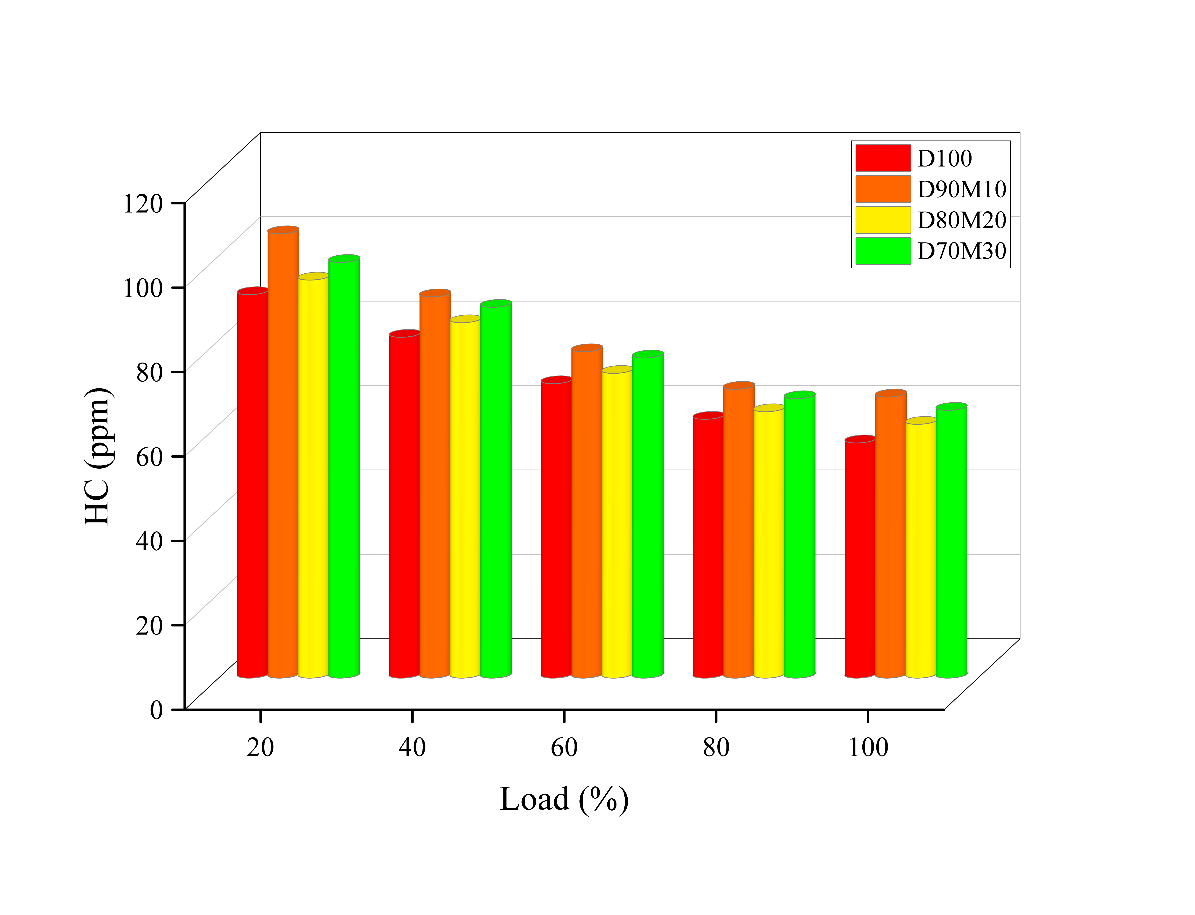


Supplementary fig 7. Impact of engine load and mahua biodiesel blend ratio on HC emission


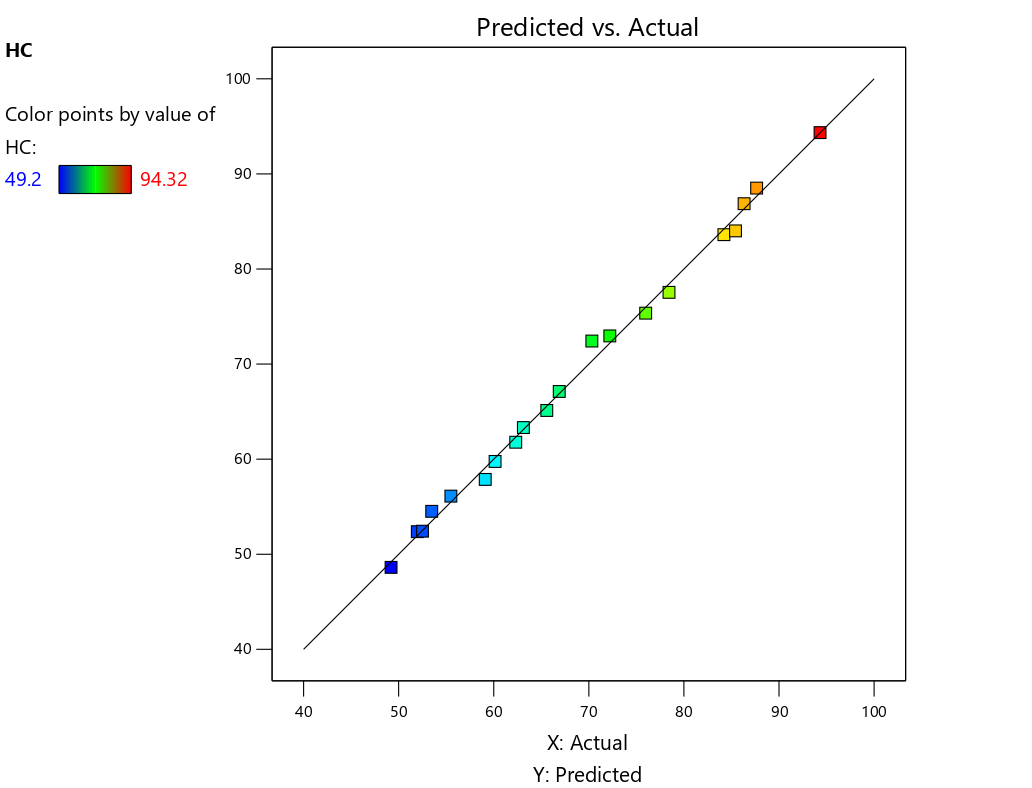


Supplementary fig 8. Actual vs predicted values of HC emission


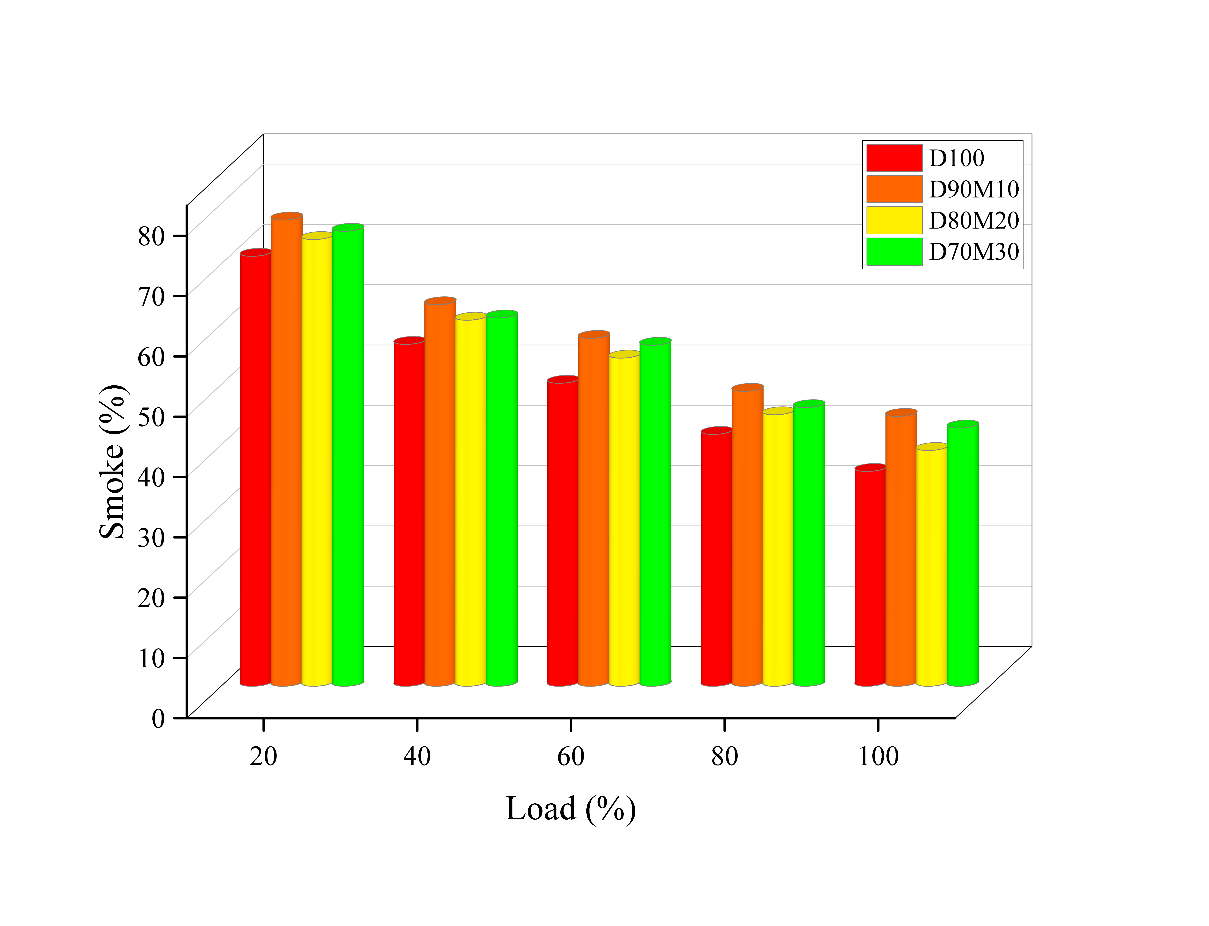


Supplementary fig 9. Impact of engine load and mahua biodiesel blend ratio on smoke emission


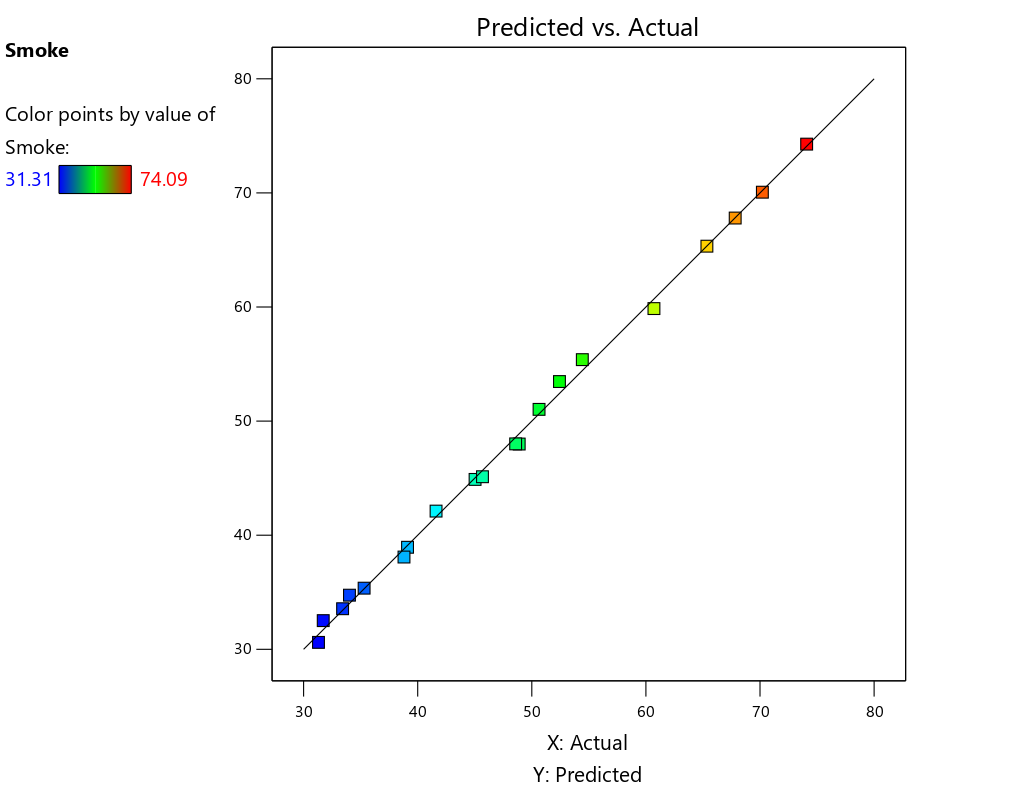


Supplementary fig 10. Actual vs predicted values of smoke emission


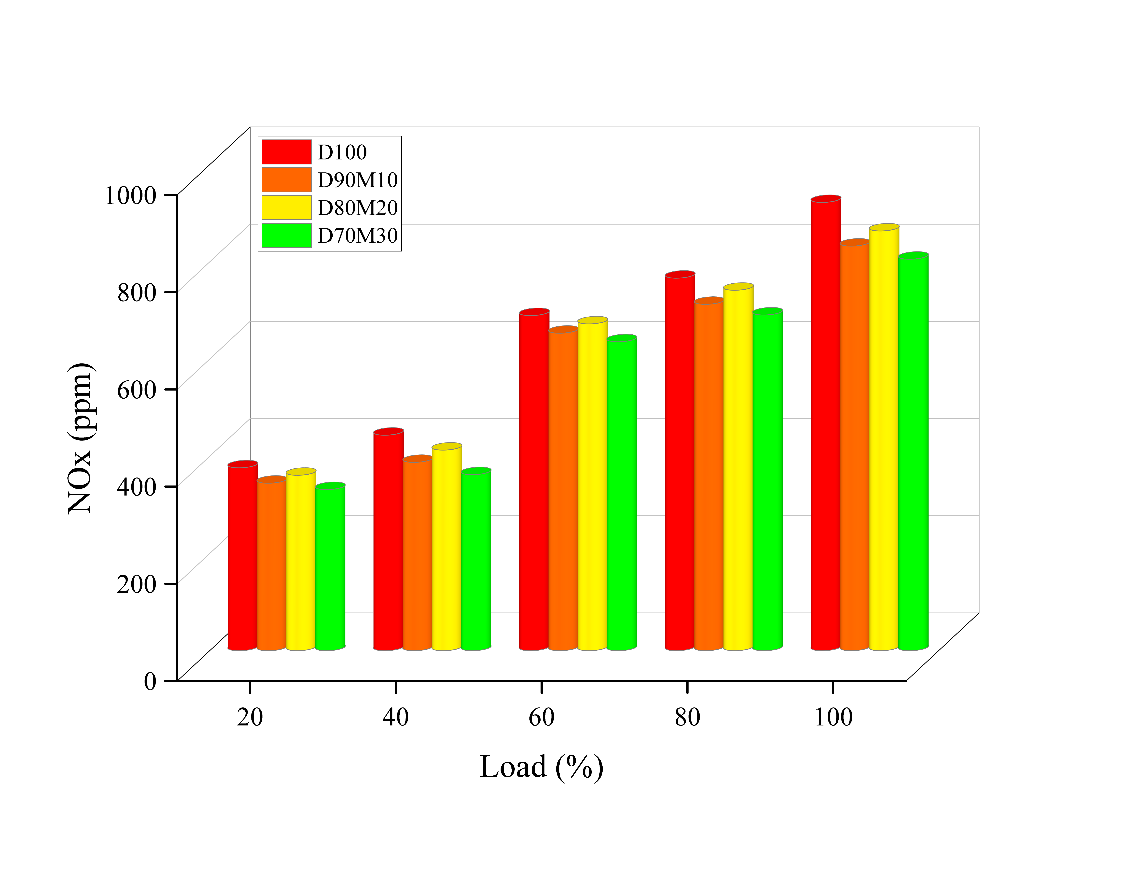


Supplementary fig 11. Impact of engine load and mahua biodiesel blend ratio on NOx emission


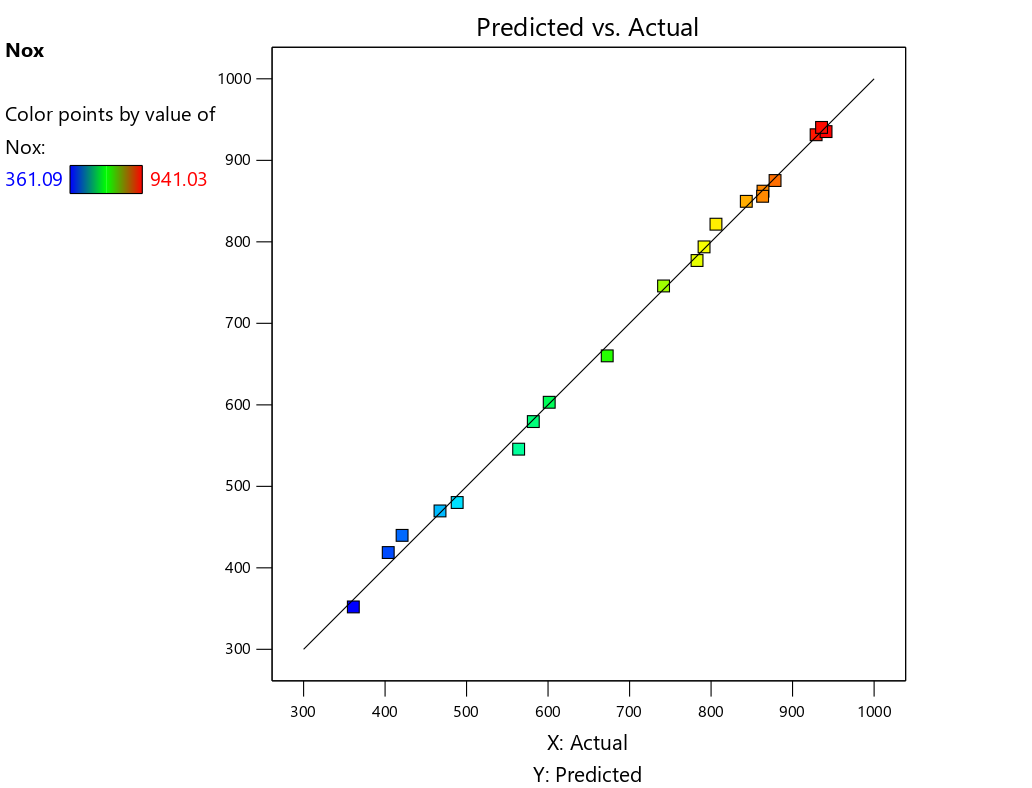


Supplementary fig 12. Actual vs predicted values of NOx emission
